# High temporal resolution of glucosyltransferase dependent and independent effects of *Clostridium difficile* toxins across multiple cell types

**DOI:** 10.1186/s12866-015-0361-4

**Published:** 2015-02-04

**Authors:** Kevin M D’Auria, Meghan J Bloom, Yesenia Reyes, Mary C Gray, Edward J van Opstal, Jason A Papin, Erik L Hewlett

**Affiliations:** Department of Biomedical Engineering, University of Virginia, PO Box 800759, Charlottesville, VA 22908 USA; Division of Infectious Diseases and International Health, Department of Medicine, University of Virginia, PO Box 801340, Charlottesville, VA 22908 USA; Current address: Vanderbilt University School of Medicine, 340 Light Hall, Nashville, TN 27232 USA

**Keywords:** *Clostridium difficile*, Toxin A, Toxin B, Glucosyltransferase, Epithelial, Endothelial

## Abstract

**Background:**

*Clostridium difficile* toxins A and B (TcdA and TcdB), considered to be essential for *C. difficile* infection, affect the morphology of several cell types with different potencies and timing. However, morphological changes over various time scales are poorly characterized. The toxins’ glucosyltransferase domains are critical to their deleterious effects, and cell responses to glucosyltransferase-independent activities are incompletely understood. By tracking morphological changes of multiple cell types to *C. difficile* toxins with high temporal resolution, cellular responses to TcdA, TcdB, and a glucosyltransferase-deficient TcdB (gdTcdB) are elucidated.

**Results:**

Human umbilical vein endothelial cells, J774 macrophage-like cells, and four epithelial cell lines (HCT8, T84, CHO, and immortalized mouse cecal epithelial cells) were treated with TcdA, TcdB, gdTcdB. Impedance across cell cultures was measured to track changes in cell morphology. Metrics from impedance data, developed to quantify rapid and long-lasting responses, produced standard curves with wide dynamic ranges that defined cell line sensitivities. Except for T84 cells, all cell lines were most sensitive to TcdB. J774 macrophages stretched and increased in size in response to TcdA and TcdB but not gdTcdB. High concentrations of TcdB and gdTcdB (>10 ng/ml) greatly reduced macrophage viability. In HCT8 cells, gdTcdB did not induce a rapid cytopathic effect, yet it delayed TcdA and TcdB’s rapid effects. gdTcdB did not clearly delay TcdA or TcdB’s toxin-induced effects on macrophages.

**Conclusions:**

Epithelial and endothelial cells have similar responses to toxins yet differ in timing and degree. Relative potencies of TcdA and TcdB in mouse epithelial cells *in vitro* do not correlate with potencies *in vivo*. TcdB requires glucosyltransferase activity to cause macrophages to spread, but cell death from high TcdB concentrations is glucosyltransferase-independent. Competition experiments with gdTcdB in epithelial cells confirm common TcdA and TcdB mechanisms, yet different responses of macrophages to TcdA and TcdB suggest different, additional mechanisms or targets in these cells. This first-time, precise quantification of the response of multiple cell lines to TcdA and TcdB provides a comparative framework for delineating the roles of different cell types and toxin-host interactions.

**Electronic supplementary material:**

The online version of this article (doi:10.1186/s12866-015-0361-4) contains supplementary material, which is available to authorized users.

## Background

*Clostridium difficile* infections, with an annual occurrence in the US of over 300,000, cause potentially fatal diarrhea and colitis [1]. These pathologies arise from the release of two potent, homologous, protein toxins—TcdA and TcdB—into the host gut. Another toxin, binary toxin, is associated with higher patient fatality rates, yet binary toxin alone is not sufficient to cause disease in animal models [2,3]. The interactions of TcdA and TcdB with many cell types lead to disease, yet the relative sensitivities and roles of different cell types remain poorly understood. Both toxins disrupt the epithelial barrier by causing epithelial cells to round and detach [4]. Neutrophil infiltration and activation of other immune cells, driven by inflammatory signals, are also key to toxin-induced enteritis [5]. Though several molecular mediators of disease have been identified, little is understood about the host cell dynamics and the role of each cell type involved [6,7]. To explore the toxins’ effects on different cells, facets of the host response have been studied using cell lines treated with TcdA and/or TcdB (e.g., release of cytokines [6,8,9], changes in cell morphology [10,11], gene expression [12,13], and cell death [14,15]). Most of these assays used in previous studies are limited to few time points, and since both toxins affect cells rapidly (in less than one hour), it is unknown if either toxin has additional effects on finer time scales and if any of these effects are consistent across cell lines at comparable concentrations.

Assays or methods that record measurements with high temporal frequency reveal small, but potentially important changes that would go unnoticed in endpoint assays. For example, live cell imaging or high-content screening methods produce near-continuous, sensitive readouts of different cellular responses. We and others have tracked temporal changes in cell morphology and attachment in response to TcdA or TcdB by continuously measuring electrical impedance across the surface of a cell culture [16-18]. In this method, cells are grown on top of a bed of electrodes covering a large portion of the surface of a well. The media completes the circuit between electrodes. When cells grow or increase their footprint or adherence, electrical current cannot as easily pass between electrodes and the electrical impedance rises. Cell rounding, shrinking, and/or death decrease impedance. It is important to note that impedance data alone does not measure one particular cell interaction (e.g., growth, adherence, spreading). Impedance is affected by a combination of many variables. However, because of the high sensitivity and precision provided, impedance data has been used as a sensitive diagnostic to detect the presence of a toxin—as a more quantitative replacement of assays that are dependent on visualization of cell rounding.

In this study, we recognize that this impedance data, in addition to indirectly detecting the amount of toxin in samples, can further be analyzed to reveal previously unrecognized, dynamic responses of host cells. Our analyses and associated metrics also allow precise comparisons between the effects of TcdA and TcdB and between different cell types. Using epithelial and endothelial cells, these analyses identify characteristics such as the minimal effective toxin concentrations and the shortest time to measurable toxin effects; standard curves with wide dynamic ranges can also be derived. Impedance changes of other cells, such as macrophages, are not as easily linked to known cell functions, but the data reveal toxin effects that would not otherwise be observed at lower temporal resolution. This understanding contextualizes the potential roles and relative abilities of different cell types to respond directly to toxin during an infection.

Impedance curves that profile cell responses also provide insight into the toxins’ molecular functions. TcdA and TcdB have glucosyltransferase domains that inactivate small GTPases. With the use of engineered mutant toxins, glucosyltransferase activity has been found necessary for cell rounding [19]. However, evidence that some glucosyltransferase-deficient mutants of TcdB (gdTcdB) are cytotoxic has raised questions about whether there are other, previously unknown toxin activities [20]. In order to identify changes dependent and independent of glucosyltransferase activity, we use gdTcdB to evaluate the dynamics of the response of macrophage and an epithelial cell line to gdTcdB. We also leverage the unique response profiles to TcdA, TcdB, and gdTcdB in order to investigate synergy or antagonism between toxins.

The cell response profiles define the dynamics of basic changes in cell physiology (e.g., cell rounding) across multiple cell types in response to TcdA, TcdB, and gdTcdB. This understanding identifies those times most representative of the entire cell response, delineates the contribution of glucosyltransferase activity to overall toxin effects, and suggests the relative roles of various cells during toxin-mediated disease.

## Methods

### Cell culture

Our experiments include HUVECs, CHO cells, HCT8 cells, T84 cells, or an immortalized, cecal, mouse epithelial cell line (hereon referred to as IMCE cells). HCT-8 cells were cultured in RPMI-1640 supplemented with 10% heat-inactivated fetal bovine serum (HI-FBS) and 1 mM sodium pyruvate. J774A.1 cells were cultured in DMEM high glucose media supplemented with 10% HI-FBS, 1 mM sodium pyruvate, and MEM nonessential amino acids (Gibco 11140). HUVEC cells (passage 3) were cultured in endothelial growth medium (EGM-Bullet Kit CC-3124, Lonza group). T84 cells were grown in an equal mixture of Ham’s F12 and Dulbecco’s modified Eagle’s media supplemented with 2.5 mM L-glutamine and 5% HI-FBS. All cells were incubated at 37°C/5% CO_2_. In our analyses, we include our previous data from IMCE cells which were derived by Becker *et al.* and incubated at 33°C as described previously [18,21]. For experiments with neutrophils (Additional file 1), procedures were approved by the University of Virginia Institutional Review Board for Health Science Research and each donor gave written consent. TcdA and TcdB, isolated and purified from strain VPI-10463, were a generous gift from David Lyerly (TECHLAB Inc., Blacksburg, VA). Recombinant gdTcdB (D286N/D288N) and TcdB were a generous gift from the laboratory of Aimee Shen. All reported results are from experiments using native TcdB. The cytopathic effects of recombinant TcdB was confirmed using HCT-8 cells (Additional file 1).

### Electrical impedance assay

Impedance was measured using the xCELLigence RTCA system (ACEA Biosciences), which consists of an RTCA DP Analyzer and 16-well E-plates. PBS was added around all wells to prevent evaporation. In each well, 100 μL media was incubated at room temperature for 30 minutes, and one baseline reading was taken. Cells in 100 μL media were then added and allowed to settle at room temperature for 30 minutes. Cells were allowed to grow directly on electrodes, without coating, in order to provide the most direct measurement of changes in cell morphology and/or adherence. Plates were then moved inside the RTCA DP Analyzer inside a CO_2_ incubator at 37°C. Subsequent readings were taken at frequencies ranging between every 4 seconds to every 10 minutes, with higher frequency measurements reserved for times directly before toxin addition to at least 6 hours after addition (complete protocols and data files available in the Additional file 1).

Since the impedance measurements are sensitive to slight movements or vibrations, the method by which toxin was added to cells was an important consideration. In our initial experiments, mechanical agitation and replacement of media sometimes caused small, sharp spikes in electrical impedance. To minimize disturbances, plates were not removed from the RTCA Analyzer once the experiment was begun. Toxins prepared in media (10x) were gently pipetted using only one or two depressions. Media was not replaced after the addition of toxin.

### Cell viability

Viability was assessed by the ability of cells to metabolize a colorless tetrazolium salt to formazen, an orange-colored product (Cell Counting Kit-8 from Dojindo Molecular Technologies). In accordance with the manufacturer’s instructions, 10 μL of CCK-8 solution was added to 100 μL wells and incubated for 1 h before measuring the absorbance at 450 nm. Viability is reported as the percentage of signal of untreated cells. Lysed cells did not produce any signal.

### Analyses

The protocols, data, computer code, and instructions for running the code that reproduce all results and figures are provided in the Additional file 1. Due to the volume of data (thousands of points measured along each of several curves), the standard deviation is visualized as shaded regions.

## Results

### Quantification of the cytopathic effects elicited by TcdA and TcdB

**Figure 1:**
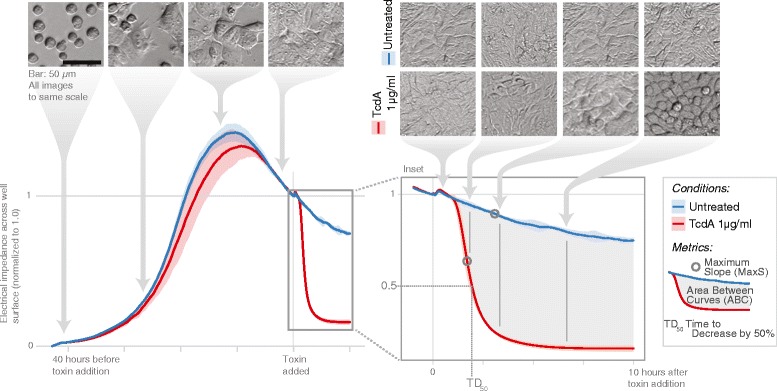
**Measurement of toxins’ cytopathic effects by tracking electrical impedance across the surface of a cell culture.** All impedance readings were divided by the impedance at the time toxin was added (normalized impedance). Shaded regions above and below lines represent the standard deviation of technical replicates (n = 2). Readings were taken as quickly as every four seconds (Methods). The brightness of each photograph was adjusted digitally (uniformly across an entire photograph) to make the overall brightness across all photographs similar.

**Figure 2:**
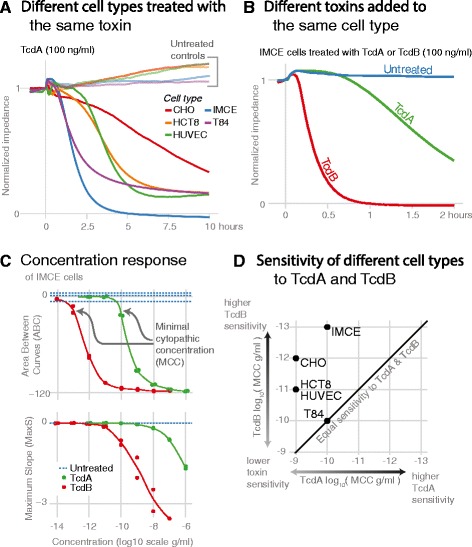
**Quantification of cytopathic effects. (A and B)** The cytopathic effects between cell types and toxins can easily be distinguished. **(C)** The impedance curves can be analyzed to produce two metrics, ABC and MaxS, which can then be used to define the minimal cytopathic concentration (MCC). **(D)** The MCC of TcdA and TcdB for five cell lines define cell line specific sensitivities.

In order to assess the cytopathic effects of TcdA and TcdB, we measured changes in impedance across the surface of electrode-embedded wells (Methods). Impedance is dependent upon cell number, adherence, and morphology. It increases as cells proliferate or spread, and it decreases when toxin is added and cells round up (Figure 1). The rate at which impedance decreases is dependent on the toxin, toxin concentration, and cell type (Figure 2A, B). To summarize the data-rich “impedance curves”, we calculated simple metrics: the area between the curves of control and toxin-treated cells (ABC, gray area in inset of Figure 1) and the maximum slope of a curve (MaxS, Figure 1). Both metrics produce log-linear calibration curves (Figure 2C). A negative ABC indicates that the impedance curves of toxin-treated cells are below the curves of untreated cells. Blue, dashed lines in Figure 2C show the variability of the ABC of control cells from their average impedance curve. Standard curves relating the time for impedance to drop by 50% (TD_50_) to toxin concentration have been generated before [22], yet we found that small experimental error in recording times at which toxin was added directly translated to errors in TD_50_. However, MaxS was more consistent between replicates. In simpler terms, for replicates within and between experiments, the time required to observe a change in impedance was more variable than the rate of that change. The other metric, ABC, captures long-term effects by integrating readings over several hours. The minimal concentration to induce an observable change in impedance from control is denoted as the minimal cytopathic concentration (MCC; Figure 2C). When ABC and MaxS are considered together, toxin concentration can be determined with a dynamic range spanning six orders of magnitude or more (depending on toxin and cell type). Together, these metrics allow for thousands of data points and hundreds of wells to be simultaneously visualized and summarized to dozens or fewer of numbers that can be easily interpreted (e.g., Figure 2D and Additional file 1).

### Epithelial and endothelial cells: similar characteristic responses but different sensitivities to TcdA and TcdB

In the first set of comparisons, we chose four well-characterized cell types or cell lines—one endothelial (HUVECs) and three epithelial (CHO, HCT8, and T84)—and one immortalized, cecal, mouse epithelial cell line (IMCE, see Methods). For these five cell lines, the MCC for TcdA and TcdB varied over ranges of 0.1-1 ng/ml and 0.1-100 pg/ml, respectively (Figure 2D). We did not find a maximal effective concentration of either toxin (1 μg/ml was the highest concentration tested). TcdB was consistently 100–1000 times more potent than TcdA, except in T84 cells, which were equally sensitive to TcdA and TcdB (as measured by MCC). The curves were largely similar in that they all consisted of a short delay followed by a sharp decrease that then leveled off (Figure 2A); differences were primarily in scale. Determining the time to the onset of the first toxin effects was complicated slightly by the physical process of adding toxins to wells—a process which caused disturbances that temporarily affected impedance (note the early “bump” in Figure 2A). Nevertheless, differences between control and toxin-treated cells can be distinguished. Across all cell types, the time required for an impedance curve to diverge from control was more than ten minutes. Nothing clearly suggested an immediate response to toxin binding. Morphological changes might not occur until after toxins enter cells and act intracellularly. We next examined early effects of toxins on macrophages and investigated the contribution of glucosyltransferase activity to the dynamics of cell responses.

### Macrophages: rapid, sensitive, complex concentration-dependent responses to TcdA and TcdB

**Figure 3:**
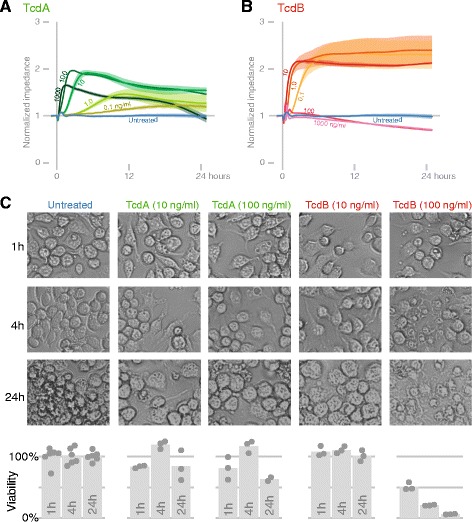
**Macrophage responses to TcdA and TcdB. (A and B)** Impedance curves from a range of TcdA and TcdB concentrations. Both graphs represent one experiment where confluent cells were treated with toxin (replicate experiments shown in Additional file 1). **(C)** Replicates experiments were performed in transparent wells for brightfield microscopy. The viability of cells as measured by the CCK-8 assay (Methods) is shown in the bar charts beneath the microscopy images.

J774 mouse macrophages were as sensitive and responsive to TcdA and TcdB as epithelial cells. The impedance of macrophages treated with TcdA (300 ng/ml) and TcdB (10 ng/ml) diverged from controls in 10 and 20 minutes, respectively (Additional file 1). In contrast to epithelial cells, however, the impedance of macrophages increased after toxin addition (Figure 3), and the responses of J774 cells to TcdA and TcdB differed in shape and scale. TcdA caused a rise in impedance at 0.1 ng/ml, and the magnitude and speed of this rise increased until TcdA concentration reached 100 ng/ml (Figure 3A). The elevated impedance correlated with spreading and protrusions from macrophages (Figure 3C). At higher concentrations (300 and 1000 ng/ml), the slope of the rise continued to increase, yet the rise was inhibited, as if stopped prematurely before reaching its peak, and then impedance dropped below that of control cells (Figure 3A and Additional file 1). This decrease at 24 h correlated with a loss of viable cells (Figure 3C). The unexplained, increased viability at 4 h may be due to altered metabolism of J774 cells. TcdB at 0.1, 1, and 10 ng/ml caused impedance to rise and stabilize at approximately double the initial value; only the slope of the rise (not the final height) was affected by toxin concentration (Figure 3B and Additional file 1). Like TcdA, cell spreading correlated with elevated impedance. Unlike TcdA, the concentrations of TcdB that caused increased impedance did not result in any loss of viable cells (Figure 3C).

At 100 and 1000 ng/ml of TcdB, the impedance curves were entirely different than lower concentrations. Instead of rising, impedance fell. This characteristic correlated with a loss of viability (Figure 3C). Hence, at or between 10 and 100 ng/ml, the response of macrophages to TcdB switches from cell stretching and increased adherence to a degradation of cell structure and loss of viability.

### TcdB glucosyltransferase activity is required for full cytopathic effects in HCT8 epithelial cells

**Figure 4:**
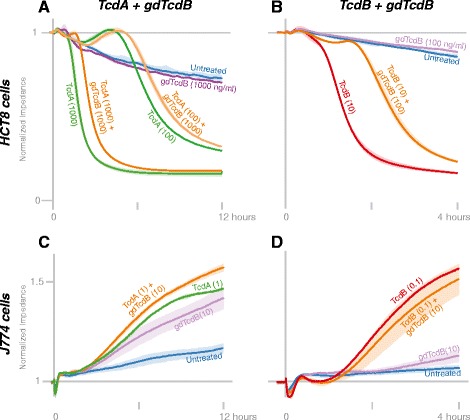
**Response of HCT8 epithelial cells and J774 macrophages to TcdA + gdTcdB, and TcdB + gdTcdB. (A-B)** Confluent HCT8 cells treated with toxins (see Additional file 1 for replicate experiments). **(C-D)** Confluent J774 cells treated with toxins (see Additional file 1 for replicate experiments).

Since the cytopathic effects of TcdA and TcdB have been attributed to their glucosyltransferase activities, we expected that gdTcdB would not cause cell rounding. Indeed, the responses of HCT8 cells treated with gdTdcB-treated (100 and 1000 ng/ml) and untreated cells were indiscernible in the first ten hours after toxin addition (Figure 4A, B). After five days, gdTcdB at 1000 ng/ml did eventually cause cytopathic effects (Additional file 1). However, it is not clear if such a slow effect is glucosyltransferase-independent or is due to residual toxin activity. Nevertheless, glucosyltransferase activity is required for the full, rapid cytopathic effect of TcdB in HCT8 cells.

### A glucosyltransferase-independent TcdB mechanism decreases macrophage viability

**Figure 5:**
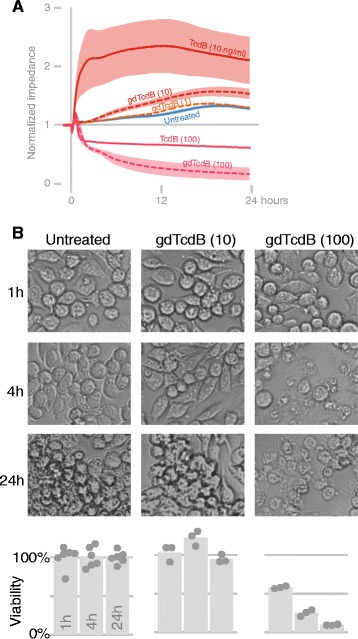
**Macrophage responses to TcdA and TcdB. (A)** Changes in impedance and of confluent J774 cells in response to gdTcdB and TcdB **(B)** A replicate experiment in transparent wells for brightfield microscopy and viability assays. Controls are the same as in Figure 3.

We next determined glucosyltransferase-dependent toxin effects on J774 macrophages. gdTcdB at 1 ng/ml did not cause macrophages to change their morphology as did TcdB (Figure 5A). At 10 ng/ml, gdTcdB slightly raises impedance, yet the overall structure and viability of cells was similar to untreated cells (Figure 5A, B). However, gdTcdB at 100 ng/ml resulted in a loss of intact and viable macrophages, similar to TcdB at 100 ng/ml (Figures 3B, C and 5B). Hence, glucosyltransferase activity is required for macrophage stretching. However, at or above 100 ng/ml, a glucosyltransferase-independent mechanism of TcdB triggers a rapid loss of viability in J774 macrophages.

### gdTcdB delays TcdA’s effects in epithelial cells

To investigate if TcdA and TcdB have overlapping activity, we performed experiments with gdTcdB plus TcdA or TcdB. We anticipated that gdTcdB would attenuate or delay the effects of TcdB and perhaps TcdA. Indeed, a tenfold excess gdTcdB delayed the onset of the effects of TcdA and TcdB in HCT8 cells (Figure 4A, B).

With J774 macrophages, gdTcdB in 100-fold excess did not clearly delay the effects of TcdB at 0.1 ng/ml (Figure 4D). In two other experiments, the average rise in impedance with gdTcdB + TcdB was delayed from the rise with TcdB alone, yet the short duration of the delay (5–15 minutes) and the variability of replicates made it difficult to definitively show that gdTcdB delays the effects of TcdB in J774 macrophages (Additional file 1). Higher gdTcdB concentrations could not be used since they would decrease cell viability (Figure 3C). gdTcdB did not delay the effects of TcdA (Figure 4C).

## Discussion

In this study we systematically profiled the dynamic responses of epithelial, endothelial, and macrophage cell lines to TcdA and TcdB, revealing relative sensitivities and complex concentration-dependent cell responses. The impedance “response profiles” provide continuous readouts representing external changes in morphology and adherence that occur from a combination of several changes within the cell. We explored how glucosyltransferase activity contributes to these changes by using gdTcdB. The response profiles raise many questions about the mechanisms for the differences we observed. Although addressing each of these in detail is beyond the scope of this study, we highlight, in the following text, the findings that bring about these questions, discuss their relevance to previous studies, and explain how they improve our current understanding of host cell responses to TcdA and TcdB.

The cytopathic effects of TcdA and TcdB are the gold standard diagnostic for infection [23,24]. However, the kinetics of these effects have typically been characterized at a limited number of time points. With a continuous assay, we were better able to observe immediate effects of toxin. Although toxins may interact immediately with the cell surface, the morphological differences (represented by impedance) occurred after a delay of ten minutes or more. Since TcdA (2.65 μg/ml) has been found to enter HT29 cells in 5–10 minutes, the delay we observed is likely because toxins must enter HCT8 cells to alter their morphology [25]. Also, in our experiments with more than one toxin, gdTcdB often delayed the onset of cytopathic effects by one hour or less. Without high temporal resolution, we would have likely missed the time window in which TcdB + gTcdB was different than TcdB alone. Hence, without precisely tracking changes in cell impedance, several molecules or proteins that delay or accelerate toxin effects could be missed.

Epithelial and endothelial cell lines had similar morphological changes, yet the rapidity of the changes distinguished different cell types, toxin concentrations, and TcdA versus TcdB. These differences could be summarized by condensing the data into metrics that represented the greatest rate of the change (MaxS) and the cumulative amount of change over several hours (ABC). When these metrics are considered together, standard curves over many orders of magnitude can be used to measure toxin concentration and determine the minimal amount of toxin necessary to induce an effect (Figure 2D). The CHO cell line was second-most sensitive to TcdB, making CHO cells a good choice for toxin detection. Indeed, a modified CHO cell line was used in the development of an ultrasensitive assay of toxin activity [16]. T84 cells, the least sensitive to TcdB, were similarly sensitive to TcdA and TcdB, as has been found previously [26]. For TcdB, the two rodent cell lines (CHO and IMCE) were more sensitive than the three human cell lines (HCT8, HUVEC, and T84), although more cell lines would be needed to confirm any species-specific sensitivity. For TcdA, cell line sensitivities were less variable than for TcdB, indicating that factors that make cells vulnerable to TcdA may be more consistent between cell lines.

Comparisons between TcdA and TcdB have often been a prominent research focus. TcdB is more cytotoxic in cell culture; TcdA is more enterotoxic in animal intoxication models [18,27]; and there are varying results about which toxin is essential for *C. difficile* infection [28,29]. By correlating differences in host cell responses to differences in disease severity, particular cell types or toxin activities can be prioritized. For instance, TcdA is more enterotoxic than TcdB in mice and hamster ceca [18,30]. This observation agrees with findings that TcdA binds more strongly than TcdB in the hamster intestine [31,32]. One might then expect that cecal epithelial cells from mice of the same genetic background as those used in the aforementioned *in vivo* studies (IMCE cells) would be more sensitive to TcdA than TcdB. Instead, IMCE cells were over 100 times more sensitive to TcdB than TcdA, suggesting that factors in addition to the cytopathic effects on epithelial cells are important in explaining the pathologies of toxins *in vivo*. The extracellular environment or other cell types may be the key mediators determining disease severity.

Previous studies have quantified the viability of either TcdA- or TcdB-treated macrophages at one or two time points [33-35]. In our results, TcdA or TcdB rapidly induced macrophages to increase adherence and/or spread. After 24 h or more of TcdA or TcdB (10 ng/ml or less) treatment, macrophages became larger and more circular. The decreased impedance with TcdA was correlated with a partial loss of viability. This result agrees in part with Melo Filo *et al.* who reported that TcdA and TcdB killed 30% and 60%, respectively, of primary mouse macrophages (1 μg/ml at 24 h) [33]. In our experiments with J774 cells, TcdB-treated cells at 10 ng/ml or less remained viable, and TcdB-treated cells at or above 100 ng/ml lossed nearly all viability. The balance of morphological changes and increased adherence versus death likely account for the complex rise and fall of impedance in toxin-treated macrophages.

TcdB caused two distinct responses in J774 macrophages: stretching (≤10 ng/ml) or a loss of viability (≥100 ng/ml). Siffert *et al.* showed TcdB-treated, human macrophages “arborize”, or stretch, with little loss of viability (1 μg/ml at 3 h and 24 h) [34]. It is possible that TcdB also causes two distinct responses in human macrophages, but Siffert *et al.* only reported results at one concentration. Although much remains to be determined about the mechanisms of these effects, early stimulation of macrophages may be associated with acute inflammation, while eventual death correlates with macrophage depletion and neutrophil accumulation in *C. difficile*-associated diarrhea [36].

The cell responses described above prompted questions about potential responses that are independent of the toxins’ glucosyltransferase activity. Only after several days were we able to observed cytotoxic or cytopathic effects of gdTcdB in HCT8 cells. Chumbler *et al.* found that glucosyltransferase mutants were cytotoxic to HeLa cells after only 2.5 h [20]. The different cell types (HCT8 versus HeLa) and different glucosyltransferase mutants may account for the differences in timing. The effects of mutant toxins have never been assessed over such long time scales with such great sensitivity.

Since TcdA and TcdB are homologous, one might expect that gdTcdB should interfere with TcdA. Indeed, in HCT8 cells, gdTcdB delayed the cytopathic effects of TcdA and TcdB. Although impedance data alone do not show that a direct or toxin-specific molecular interaction is responsible for the delay, one possible interpretation of this result is that TcdA and TcdB compete for cell entry. However, two studies using truncated toxins found that the C-termini of each toxin (believed to be necessary for internalization) do not inhibit internalization of the other toxin [37,38]. Since the glucosyltransferase domains of TcdA and TcdB share substrates, another possible interpretation is that the toxins compete after internalization. Indeed, blocking glucosyltransferase activity would reduce the cytopathic effects as is shown with gdTcdB. However, as described below, glucosyltransferase activity may not be required for all toxin effects in all cell types.

Since macrophages detect a variety of antigens, one might expect that the responses to toxin might not be entirely dependent on glucosyltransferase activity. The stretching of macrophages with TcdB concentrations at or below 10 ng/ml required glucosyltransferase activity. However, TcdB at or above 100 ng/ml destroyed macrophage structure by an unknown, glucosyltransferase-independent mechanism.

The high sensitivity of epithelial cells, endothelial cells, and macrophages to TcdA and TcdB suggests they could be damaged by direct toxin interaction in the host. However, the location of toxin during infection is poorly understood. With sensitivities of cells reaching as low as 100 pg/ml, tracking toxins by immunohistochemistry is technically challenging [32]. Assessing sensitivities *in vitro* provides an indirect measure of the roles of cell types in isolation. Additionally, TcdA and TcdB initiate a cascade of deleterious events involving multiple cells. Neuronal signals have been implicated in beginning the disease process, stimulating mast cells or macrophages that may then recruit other cells [39-42]. Neutrophil infiltration is a hallmark of intoxication, yet neutrophils *in vitro* require much higher toxin concentrations to be recruited (>1 μg/ml) [43-45]. To confirm the low sensitivity of neutrophils, we attempted to measure impedance changes of neutrophils in response to toxins, yet the variability in these primarily non-adherent cells was too high to identify differences (Additional file 1). Elements of the toxin responses of other cell types (e.g., mast cells [46-48], dendritic cells [49,50], neurons [51,52], fibroblasts [53,54], etc.) have been studied, yet the dynamics of their responses—and in some cases concentration-dependent effects—are unknown. In the future, precisely capturing the time and concentration-dependent responses to TcdA and TcdB will better contextualize their potential roles in the host.

## Conclusions

Our analyses of endothelial cells, epithelial cells, and macrophages in the same experimental framework set a precedent for high-temporal resolution comparisons of the effects of *Clostridium difficile* TcdA and TcdB. We precisely determine the relative sensitivity of various epithelial cell lines, showing an overall greater TcdB sensitivity with few exceptions. Interestingly, an immortalized mouse epithelial cell line is more sensitive to TcdB although mice injected with toxin are more sensitive to TcdA. Using gdTcdB, we found that glucosyltransferase activity is necessary for the rapid cytopathic effects of HCT8 epithelial cells or the rapid spreading of J774 macrophage-like cells. However, the ability of gdTcdB to decrease J774 cell viability indicates a glucosyltransferase-independent mechanism contributes to cell death. Additionally, responses of J774 cells to TcdA and TcdB were characteristically different, suggesting important differences in toxin mechanism and/or targets in these cells. In the future, the framework and simple analyses in this study may also be used to investigate synergy, antagonism, or interactions between bacterial toxins and other host factors that affect cells over a wide range of time scales.

## Additional file

Additional file 1:
**Supplement.**
