# Supplementary material for: High temporal resolution of glucosyltransferase dependent and independent effects of Clostridium difficile toxins across multiple cell types
Source: BMC Microbiol. 2015 Feb 4;15(1):7. doi: 10.1186/s12866-015-0361-4 (PMC4323251; doi:10.1186/s12866-015-0361-4)
Supplement: Additional file 1: — Supplement. [file 12866_2015_361_MOESM1_ESM.pdf]

Supplement

Kevin M. D'Auria[1], Meghan J. Bloom[2], Yesenia Reyes[2], Mary C. Gray[2], Edward J. van Opstal[2], Jason A. Papin[1], and Erik L. Hewlett[2]

[1]Department of Biomedical Engineering
[2]Division of Infectious Diseases and International Health, Department of Medicine
University of Virginia, Charlottesville, VA

Contents

1 Introduction **1**

2 References from manuscript **2**
2.1 Reference 1 2
2.2 Reference 2 2
2.3 Reference 3 3
2.4 Reference 4 3
2.5 Reference 5 3

3 Reproducing Figures **3**
3.1 Figure 1 3
3.2 Figure 2 5
3.3 Figure 3 6
3.4 Figure 4 7
3.5 Figure 5 7

4 Exploring the data **8**
4.1 HCT8 cells 9
4.2 CHO cells 13
4.3 IMCE cells 13
4.4 HUVECs 14
4.5 T84 cells 14
4.6 J774 cells 15
4.7 PMN leukocytes 21
4.8 Plate Layouts 24

1 Introduction

This document includes the supplemental data referred to in the manuscript as well as instructions for how to reproduce our analyses and explore the data further. Use the clickable table of contents to navigate quickly. This is a functional document in that scripts (written in the R programming languange with the `knitr` package) are embedded, and they were run during PDF creation to produce the figures and output shown. Thus, the analyses can be repeated by downloading the source of this document (with the data) or copy-pasting all of the code into one's own R console.

Most of the functions for processing this data are in the `wellz` R package, available at https://github.com/kdauria/wellz. All other necessary code and supplemental files are wrapped in an R package, called `imTox`, available at https://github.com/kdauria/imTox. In this package, the spreadsheet denoting the compounds and concentrations in each well are in `Annotations.csv` and all of the raw data files are in the `extdata/Data` directory.

```
library(devtools)
install_github("kdauria/wellz")
install_github("kdauria/imTox")
library(wellz)
```

```
library(imTox)
wells = load_data()
```

2 References from manuscript

2.1 Reference 1

"The impedance curves of cells treated with TcdA (300 ng/ml) and TcdB (10 ng/ml) diverged from controls in 10 and 20 minutes, respectively (Supplement)."

```
figure_s1(wells)
```

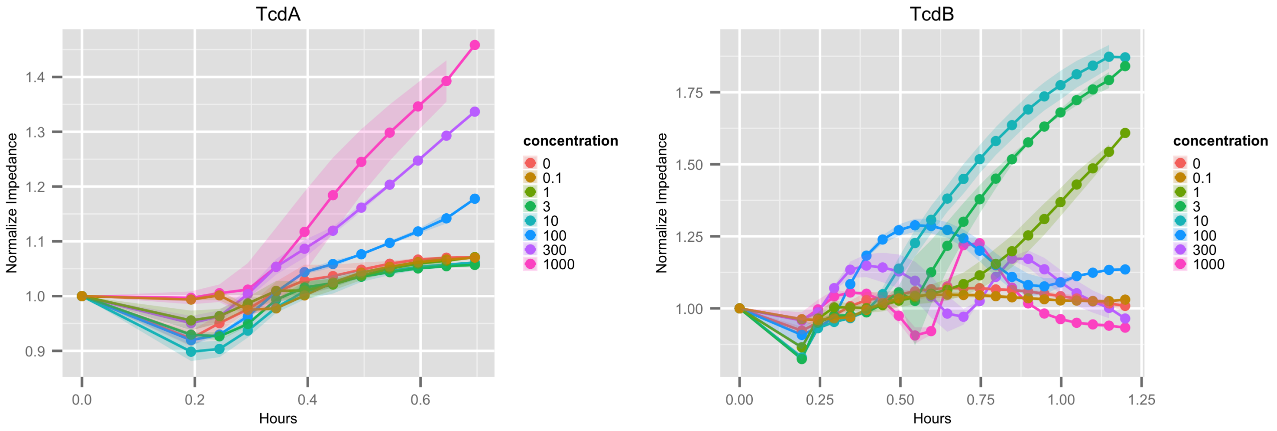

Figure S1: Early response of J774 cells to TcdA and TcdB

2.2 Reference 2

"After five days, gdTcdB at 1000 ng/ml did eventually cause cytopathic effects (Supplement)."

```
subset = normalize_toxin(select(wells, file = "HCT8-4.txt"))
plot(select(subset, "gdTcdB & !(TcdA | TcdB)"), xlim = c(-1, 150))
```

In parallel, cells were seeded in a transparent plate and imaged. The above code produces the impedance curves in Figure S2.

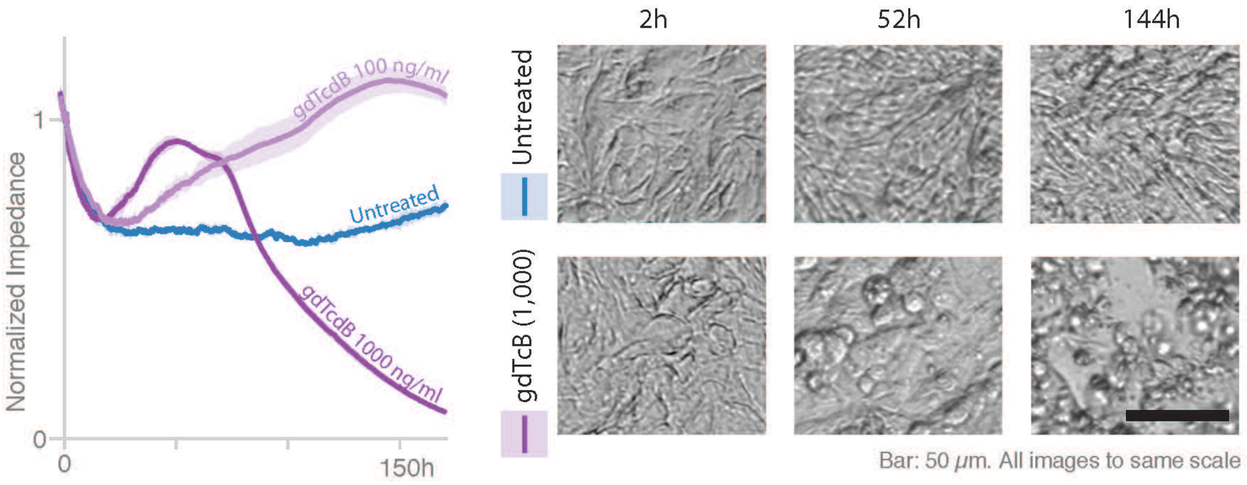

Figure S2: gdTcdB added to HCT8 cells

2.3 Reference 3

"In two other experiments, the average rise in impedance with gdTcdB+TcdB was delayed from the rise with TcdB alone, yet the short duration of the delay (5-15 minutes) and the variability of replicates made it difficult to definitively show that gdTcdB delays the effects of TcdB in J774 macrophages (Supplement). "

See Figure S21, Figure S24, and Figure S25. Figure S21 is the experiment shown in Figure 4 of the manuscript.

2.4 Reference 4

"To confirm the low toxin-sensitivity of neutrophils, we did attempt to measure impedance changes of neutrophils in response to toxins, yet the variability in these primarily non-adherent cells (impedance largely measures adherence) was too high to identify differences (Supplement)"

See subsection 4.7 for four experiments with PMNs and corresponding text.

2.5 Reference 5

"All reported results are from experiments using native TcdB. The cytopathic effects of recombinant TcdB was confirmed using HCT-8 cells (Supplement)."

The native toxin (TcdB) was more potent than the recombinant toxin (rTcdB). However, the similar changes in impedance caused by TcdB and rTcdB confrm the functionality of rTcdB.

```
subset = select(wells, "TcdB[1-1000] | rTcdB[1-1000]", file = "rHCT8.txt")
nsubset = normalize_toxin(subset)
concs = group(subset, "concentration", compound = "TcdB") + group(subset, "concentration",
    compound = "rTcdB")
plot(nsubset, color = as.character(concs), linetype = "compound", replicates = FALSE) +
    scale_colour_discrete(name = "ng/ml")
```

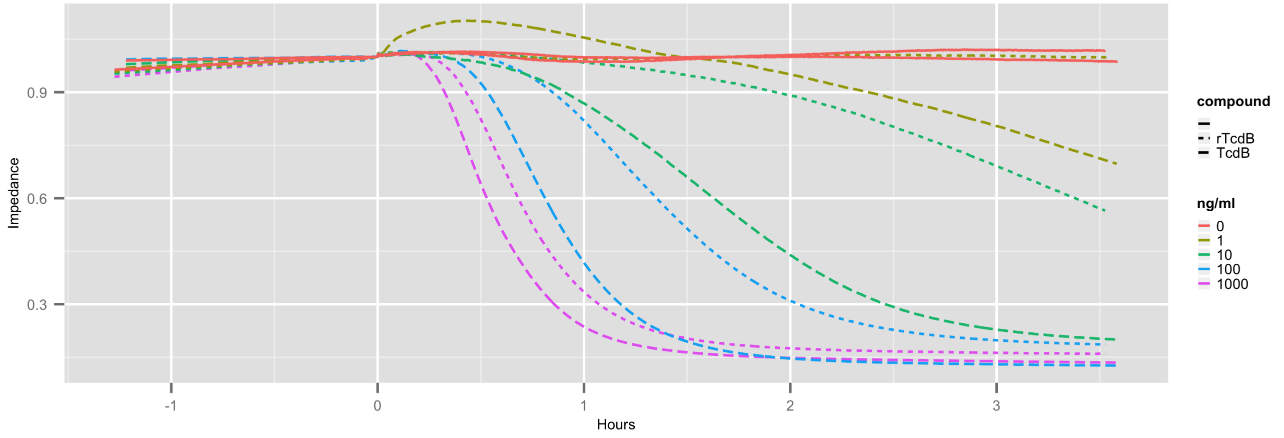

3 Reproducing Figures

Below are the functions to reproduce the figures. The displayed figures below were exported to PDFs. Cosmetic alterations (colors, line widths, axes labeling, legends, etc.) were made with Adobe Illustrator. The code for the functions can be viewed by downloading the `imTox` R package, or the code can be viewed directly in a web browser on the package's Github page. The code and package is well documented, describing any data processing (e.g., smoothing, interpolation, etc.).

3.1 Figure 1

```
figure_1(wells)
```

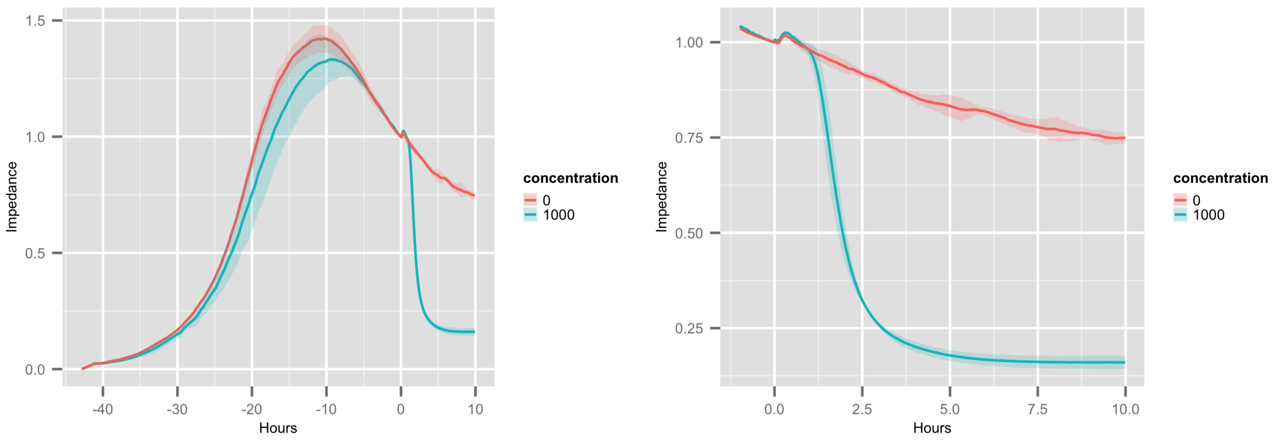

Figure S3: Plots for Figure 1 of the manuscript

3.2 Figure 2

```
figure_2(wells)
```

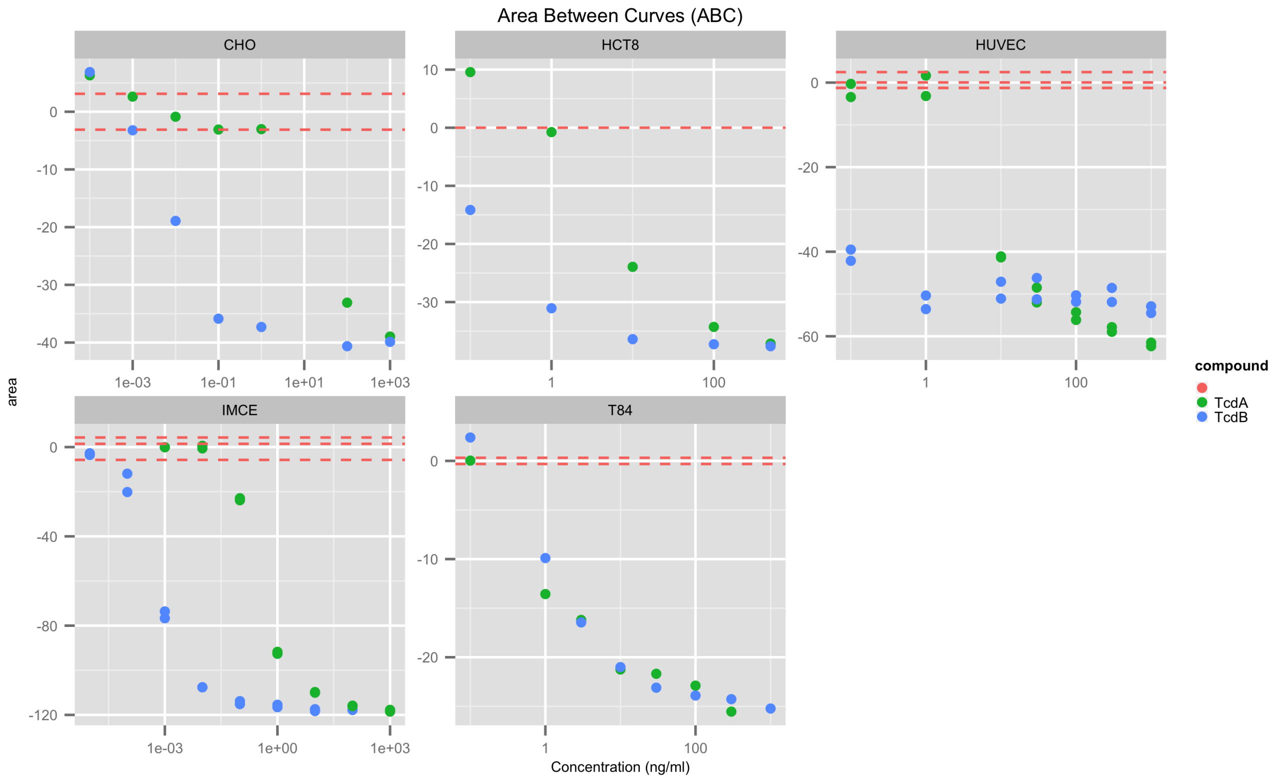

Figure S4: Plots for Figure 2 of the manuscript

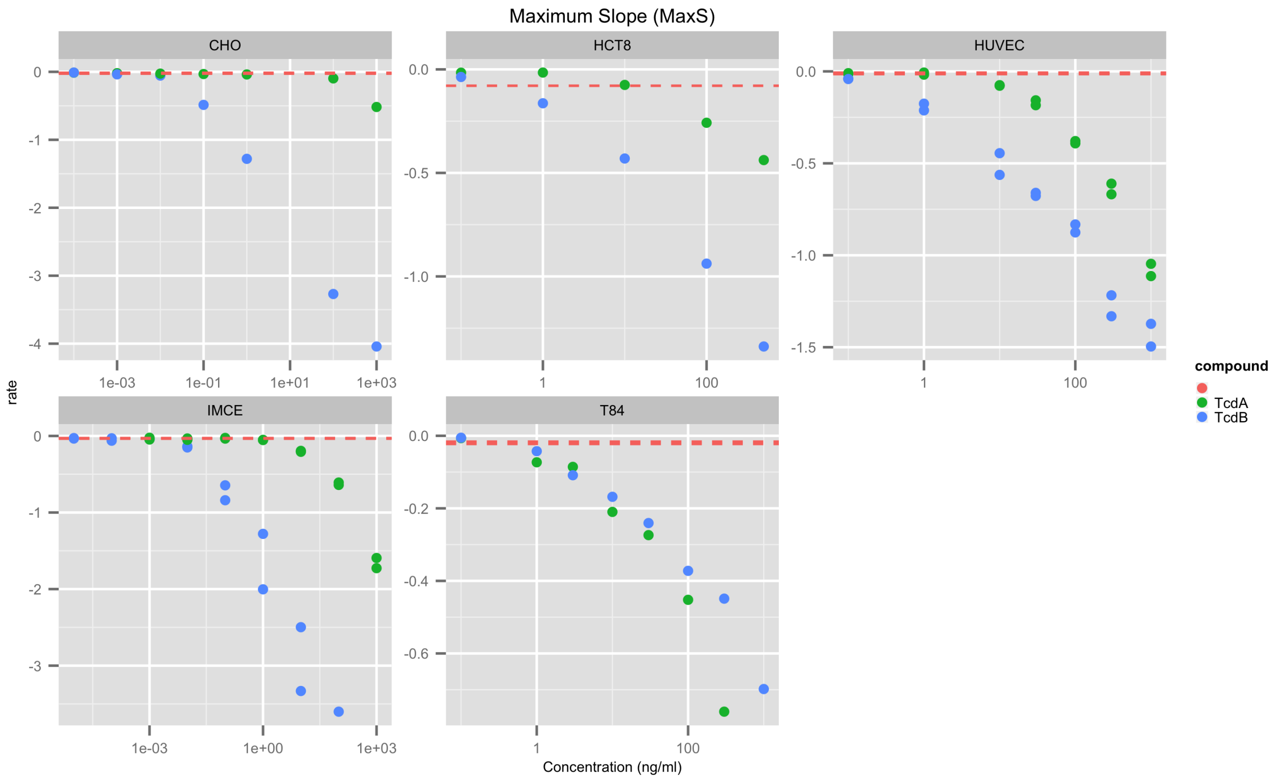

Figure S5: Plots for Figure 2 of the manuscript

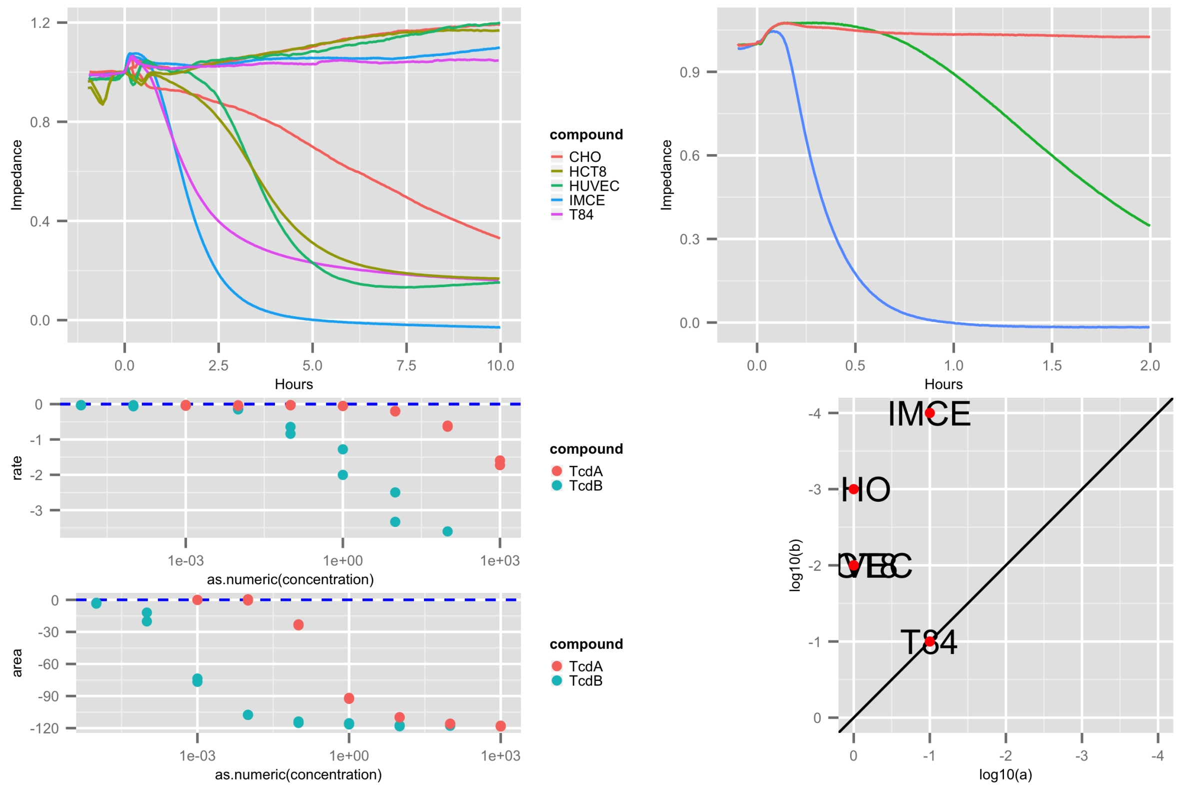

Figure S6: Plots for Figure 2 of the manuscript

3.3 Figure 3

```
figure_3(wells)
```

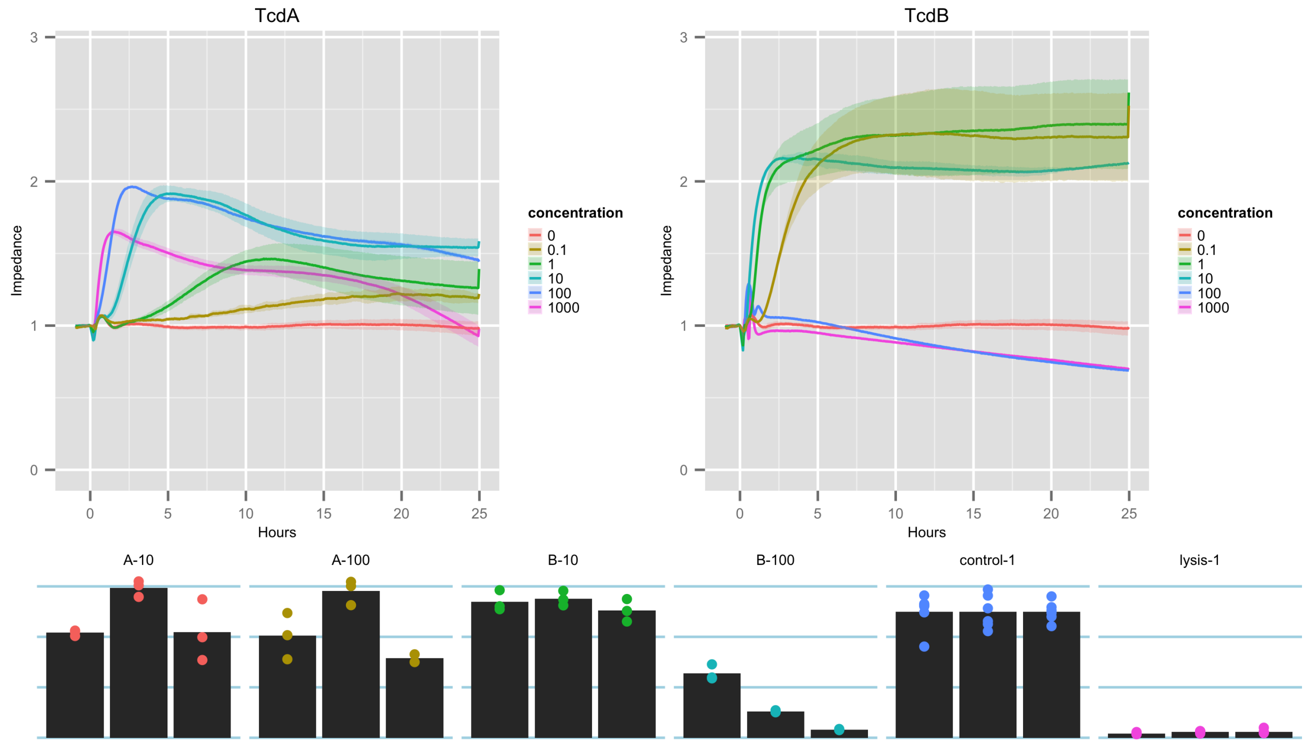

Figure S7: Plots for Figure 3 of the manuscript

3.4 Figure 4

```
figure_4(wells)
```

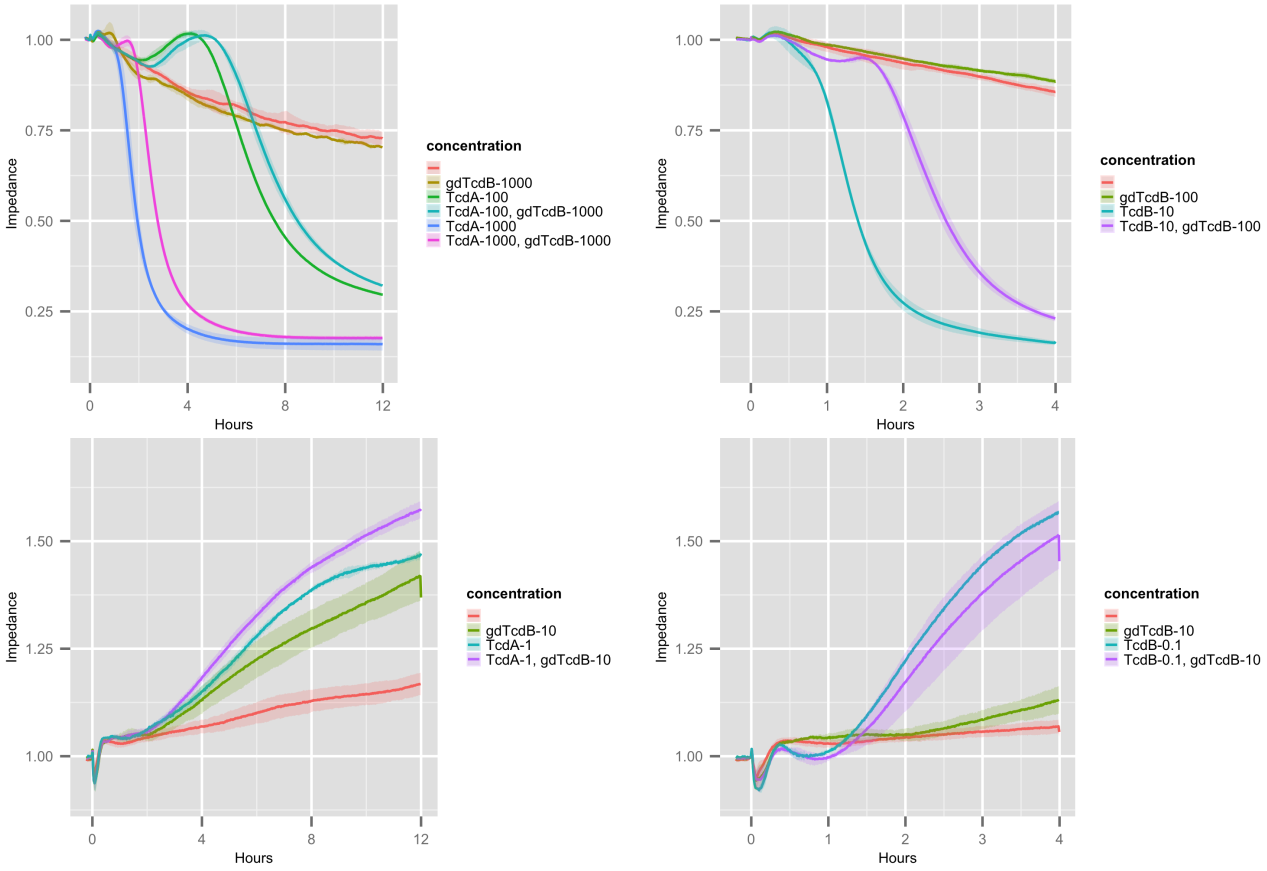

Figure S8: Plots for Figure 4 of the manuscript

3.5 Figure 5

```
figure_5(wells)
```

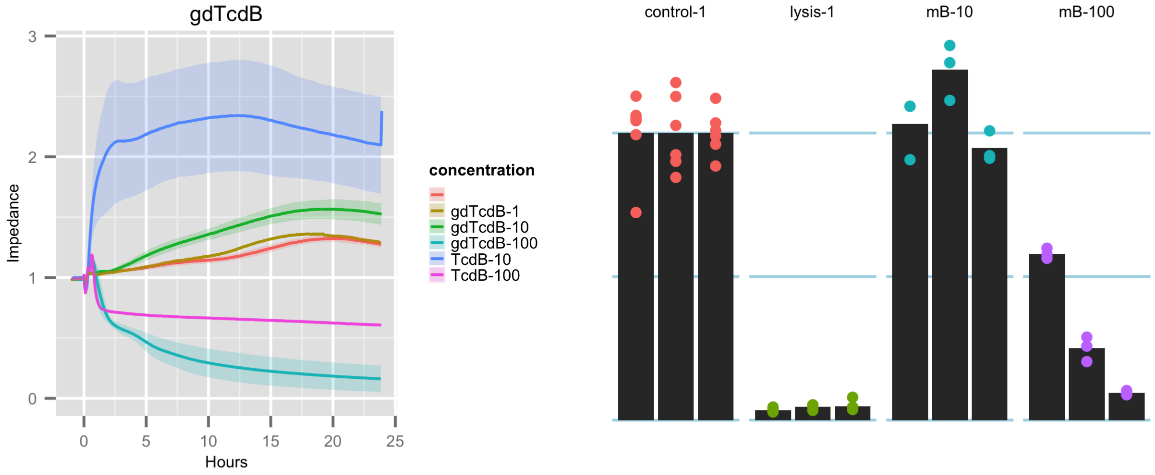

Figure S9: Plots for Figure 5 of the manuscript

4 Exploring the data

All experiments, each involving several experimental conditions, are summarized in the table below. Independent experiments that occurred on different days are separated by horizontal lines. Diagrams of the physical multi-well plates are displayed in 4.8. The number of cells seeded and all the incubation times can be found in the csv annotation file in the supplemental data, in the Table 1, or can be accessed in the `wells` variable within R (as shown in 3).

Cell type	File names	Cells	Toxins (ng/ml)	Notes
HCT8	HCT8.txt	5000	A (500, 100, 10, 1, 0.1)<br>B (500, 100, 10, 1, 0.1)	
	HCT8-2a.txt<br>HCT8-2b.txt	60000	B (1), A (100)<br>gdTcdB (100, 1000)<br>B (1) + gdTcdB (100)<br>B (1) + gdTcdB(1000)<br>A (100) + gdTcdB (1000)	toxins + gdTcdB
	HCT8-3.txt	45000	B (1), A (100)<br>gdTcdB (10, 100, 1000)<br>B (1) + gdTcdB (10)<br>B (1) + gdTcdB (100)<br>A (100) + gdTcdB (1000)	toxins + gdTcdB
	HCT8-4.txt	55000	B (10, 100), A (100, 1000)<br>gdTcdB (100, 1000)<br>B (10) + gdTcdB (100)<br>B (10) + gdTcdB (1000)<br>B (100) + gdTcdB (100)<br>B (100) + gdTcdB (1000)<br>A (10) + gdTcdB (100)<br>A (100) + gdTcdB (100)<br>A (100) + gdTcdB (1000)<br>A (1000) + gdTcdB (1000)	toxins + gdTcdB
	rHCT8.txt	60000	B (1000, 100, 10, 1, 0.1, 0.01, 0.001), A (100)<br>recombinant B (1000, 100, 10, 1, 0.1, 0.01, 0.001)	native + recombinant TcdB
CHO	CHO.txt	9000	A (1000, 100, 1, 0.1, 0.01, 1e-3, 1e-4)<br>B (1000, 100, 1, 0.1, 0.01, 1e-3, 1e-4)	
IMCE	IMCE.txt	30000	A (1000, 100, 10, 1, 0.1, 0.01, 0.001)<br>B (1000, 100, 10, 1, 0.1, 0.01, 0.001)	
HUVEC	HUVEC-a.txt<br>HUVEC-b.txt	5000	A (1000, 300, 100, 30, 10, 1, 0.1)<br>B (1000, 300, 100, 30, 10, 1, 0.1)	
T84	T84-a.txt<br>T84-b.txt	30000	A (300, 100, 30, 10, 3, 1, 0.1)<br>B (1000, 300, 100, 30, 10, 3, 1, 0.1)	
J774	J774-a.txt<br>J774-b.txt	50000	A (0.1, 1, 3, 10, 100, 300, 1000)<br>B (0.1, 1, 3, 10, 100, 300, 1000)	
	J774-2.txt	50000	B (0.00001, 0.0001, 0.001, 0.01, 0.1, 1, 10)	
	J774-3a.txt<br>J774-3b.txt	50000	A (10), B (0.01)<br>gdTcdB (0.1, 1, 100)<br>B (0.01) + gdTcdB (1)<br>B (0.01) + gdTcdB (0.1)<br>A (10) + gdTcdB (100)	toxins + gdTcdB
	J774-4.txt	50000	A (3, 300), B (1, 100)<br>gdTcdB (1, 100)<br>A (10) + gdTcdB (100)	toxins + gdTcdB
	J774-5.txt	50000	A (1, 1000),<br>gdTcdB (10, 100)<br>A (1) + gdTcdB (10)<br>A (1000) + gdTcdB (1000)<br>B (10) + gdTcdB (100)	toxins + gdTcdB
	J774-6.txt	50000	A (0.1, 1, 10, 100, 1000),<br>B (0.1, 1, 10, 100, 1000),<br>gdTcdB (1, 10, 100),<br>A (1) + gdTcdB (10)<br>B (0.1) + gdTcdB (1)	toxins + gdTcdB
PMN	PMN-a.txt<br>PMN-b.txt	360000	A (10000, 7000, 5000, 3000, 1000, 500, 100)<br>B (10000, 7000, 5000, 3000, 1000, 500, 100)	toxins + IL8
	PMN-2a.txt<br>PMN-2b.txt	360000	A (1000, 100, 10, 1, 0.1, 0.01, 0.001)<br>B (1000, 100, 10, 1, 0.1, 0.01, 0.001)	toxins alone
	PMN-3.txt	350000	A (10, 100, 1000)<br>B (1, 10, 100, 1000)	toxins alone and<br>toxins + IL8
	PMN-4.txt	350000	A (10, 100, 1000)<br>B (10, 100, 1000)	toxins alone and<br>toxins + IL8

Table 1: Experimental conditions

4.1 HCT8 cells

First, a titration was performed to determine the number of HCT8 cells needed to reach maximum impedance in a specified amount of time.

```
subset = select(wells, file = "HCT8.txt")
plot(subset, xlim = c(0, 48), type = "total", ID = "cellSeed", sd = FALSE)
```

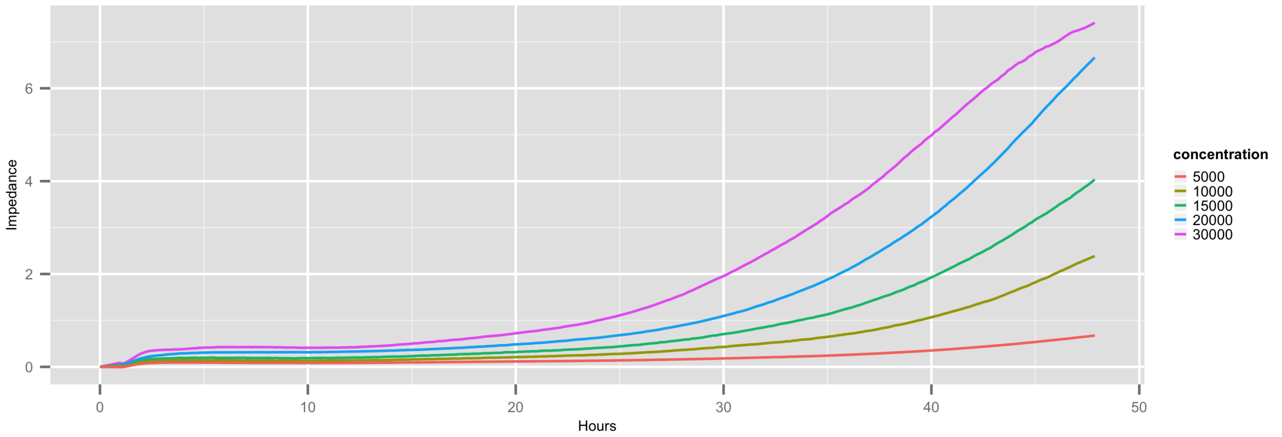

Figure S10: HCT8 cell number titration

In the same experiment, the wells seeded with 5,000 cells were used for a concentration-response experiment with TcdA and TcdB.

```
A = normalize_toxin(select(subset, "HCT8[5000] & TcdA"))
B = normalize_toxin(select(subset, "HCT8[5000] & TcdB"))
plotA = plot(A, xlim = c(-1, 10), title = "TcdA")
plotB = plot(B, xlim = c(-1, 10), title = "TcdB")
require(gridExtra)
grid.arrange(plotA, plotB, nrow = 1)
```

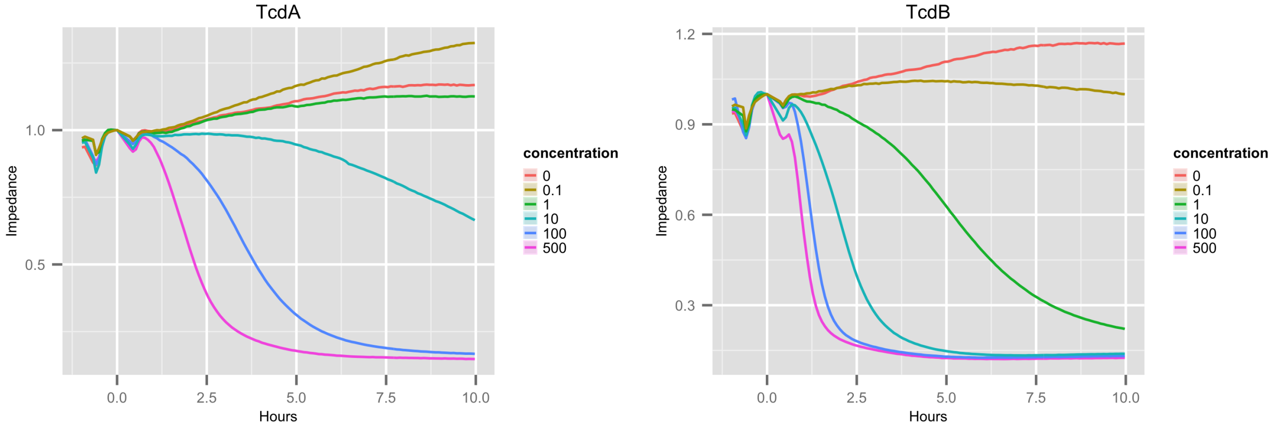

Figure S11: HCT8 cells treated with TcdA & TcdB

In the next experiment, gdTcdB or TcdA+gdTcdB and TcdB+gdTcdB combinations were added to HCT8 cells. gdTcdB had little effect within the first ten hours, yet delayed the effects of TcdA and TcdB.

```
library(ggplot2, pos = 4)
subset = select(wells, file = c("HCT8-2a.txt", "HCT8-2b.txt"))
subset = normalize_toxin(subset, xlim = c(-1, 10))
tox_plot = function(...) plot(...) + ylim(c(0, 1.2))
p1 = tox_plot(select(subset, "gdTcdB & !(TcdA | TcdB)"), title = "gdTcdB")
p2 = tox_plot(select(subset, "TcdA"), title = "TcdA")
p3 = tox_plot(select(subset, "TcdB"), title = "TcdB")
grid.arrange(p1, p2, p3, nrow = 2)
```

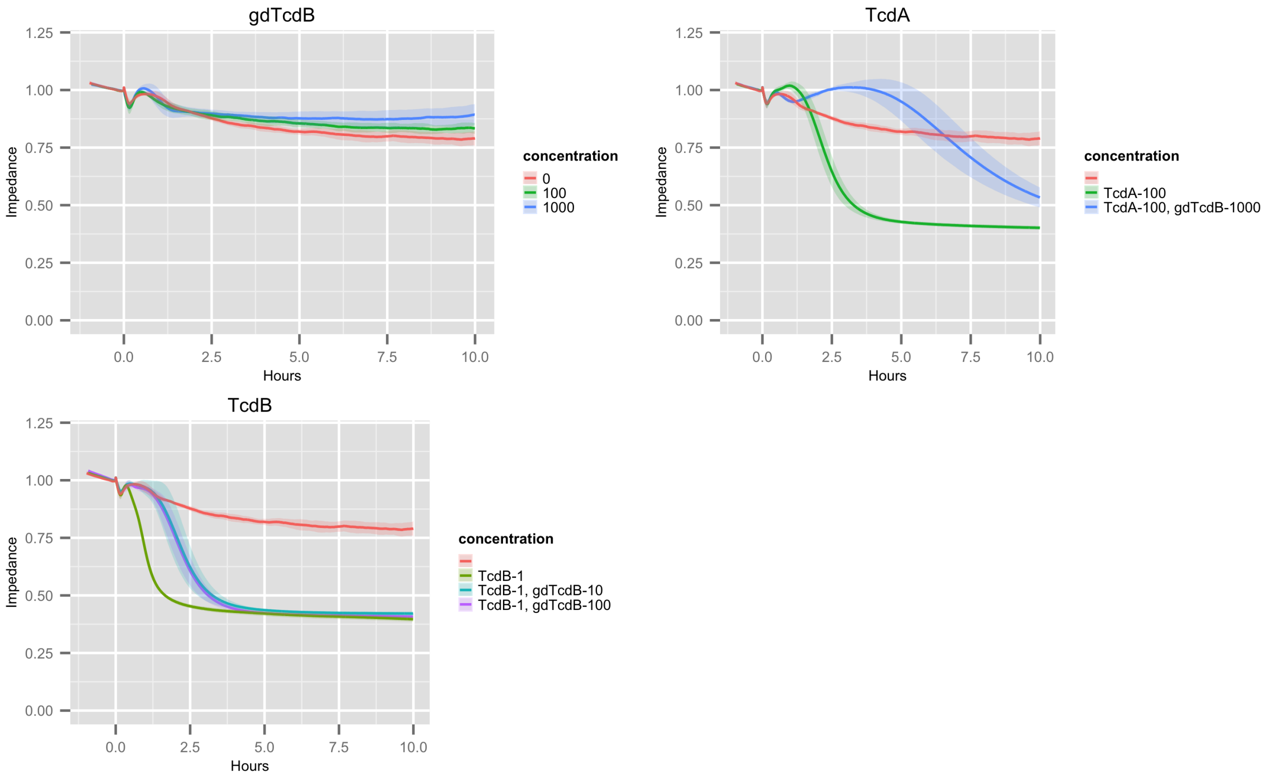

Figure S12: HCT8 cells treated with TcdA, TcdB, and/or gdTcdB

The experiment was repeated again approximately one year later, yet the variance of the growth of cells was high, presumably because of different numbers of seeded cells in each well (note the last three panels where the replicates are not averaged). This variance made it difficult to detect any potential gdTcdB-induced delay of TcdA or TcdB's effects.

```
subset0 = select(wells, "TcdA | TcdB | gdTcdB", file = "HCT8-3.txt")
subset = normalize_toxin(subset0, xlim = c(-1, 10))
p1 = tox_plot(select(subset, "!TcdA & !TcdB"), title = "gdTcdB")
p2 = tox_plot(select(subset, "TcdA"), title = "TcdA")
p3 = tox_plot(select(subset, "TcdB"), title = "TcdB")
p4 = plot(subset0, replicates = FALSE, xlim = c(0, 50))
p5 = tox_plot(select(subset, "TcdA"), replicates = FALSE, title = "TcdA")
p6 = tox_plot(select(subset, "TcdB"), replicates = FALSE, title = "TcdB")
grid.arrange(p1, p2, p3, p4, p5, p6, nrow = 3)
```

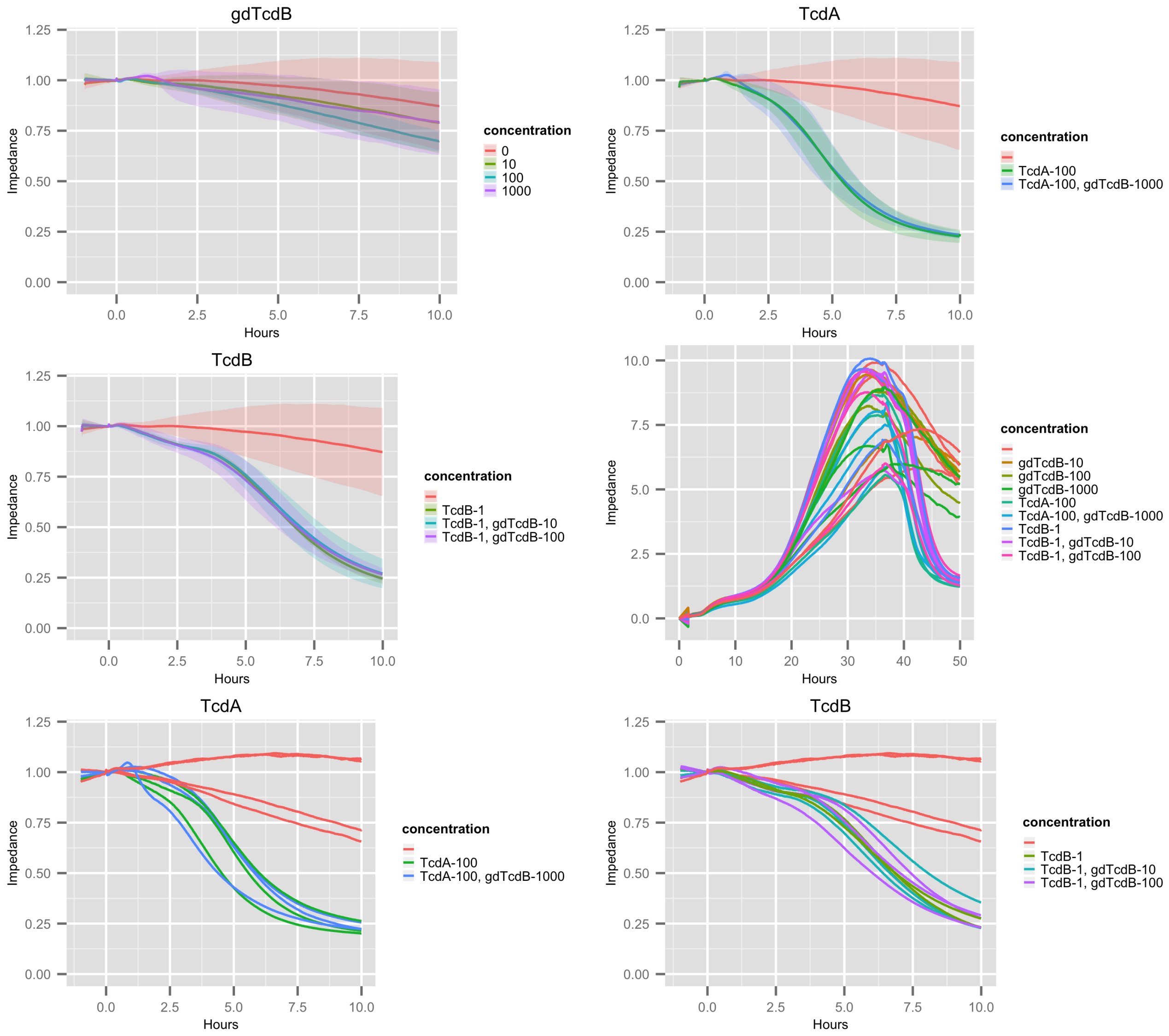

Figure S13: HCT8 cells treated with TcdA, TcdB, and/or gdTcdB

Therefore, a similar experiment was again performed. The variance among replicates was much tighter, and gdTcdB did indeed delay the effects of TcdA and TcdB. However, at the higher concentration of TcdB (100 ng/ml), this delay was not obvious; only one of the four samples showed a delay. The reason for this outlier is unclear.

```
subset = normalize_toxin(select(wells, file = "HCT8-4.txt"), xlim = c(-1, 12))
p1 = tox_plot(select(subset, "gdTcdB & !(TcdA | TcdB)"), title = "gdTcdB")
p2 = tox_plot(select(subset, "TcdA[100]"), title = "TcdA 100 ng/ml")
p3 = tox_plot(select(subset, "TcdA[1000]"), title = "TcdA 1000 ng/ml")
p4 = tox_plot(select(subset, "TcdB[10]"), title = "TcdB 10 ng/ml")
p5 = tox_plot(select(subset, "TcdB[100]"), title = "TcdB 100 ng/ml")
p6 = tox_plot(select(subset, "TcdB[100]"), replicates = FALSE, title = "TcdB 100 ng/ml")
grid.arrange(p1, p2, p3, p4, p5, p6, nrow = 3)
```

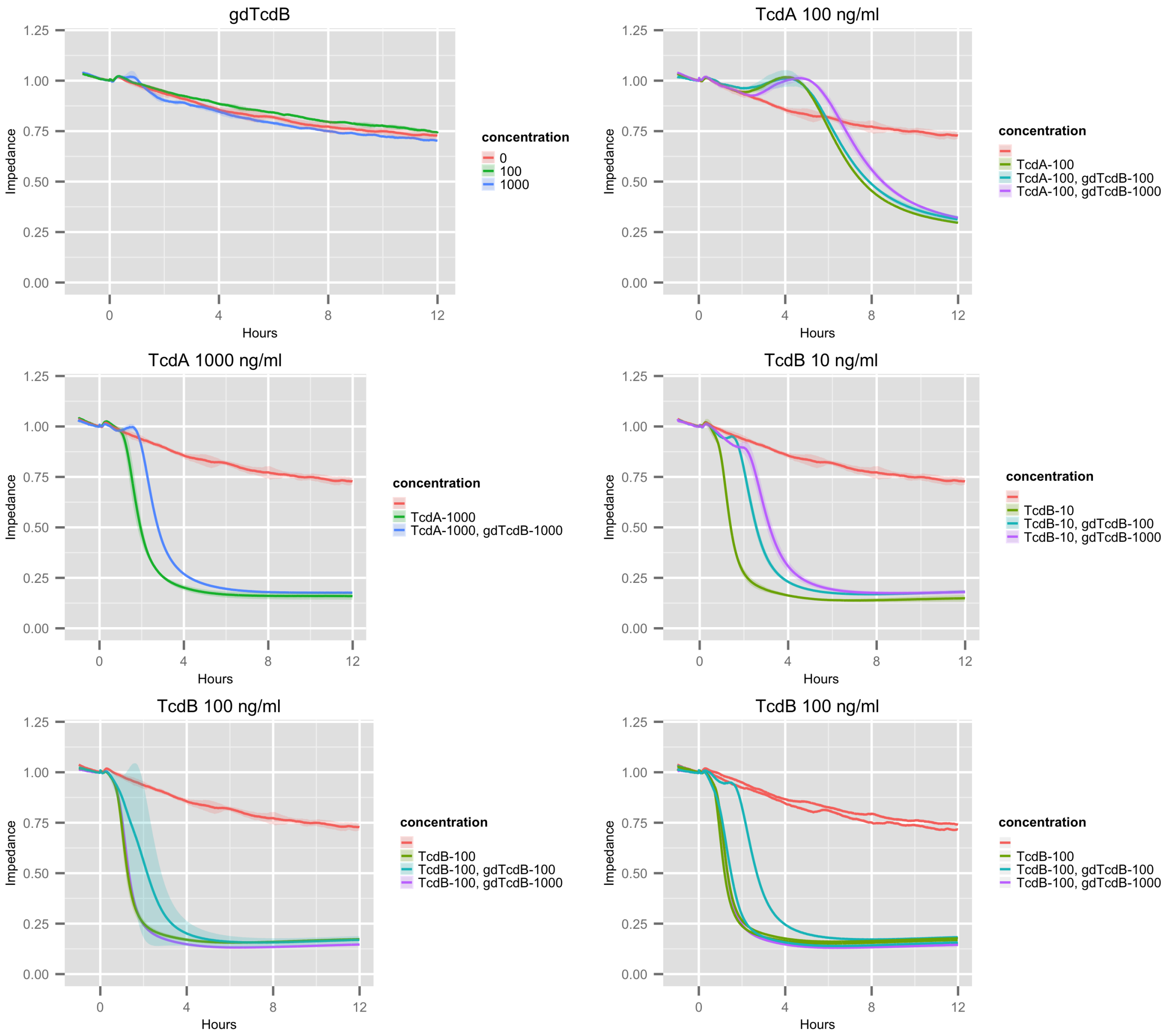

Figure S14: HCT8 cells treated with TcdA, TcdB, and/or gdTcdB

4.2 CHO cells

```
subset = normalize_toxin(select(wells, file = "CHO.txt"), xlim = c(-1, 20))
p1 = plot(select(subset, "TcdA"), title = "TcdA")
p2 = plot(select(subset, "TcdB"), title = "TcdB")
grid.arrange(p1, p2, nrow = 1)
```

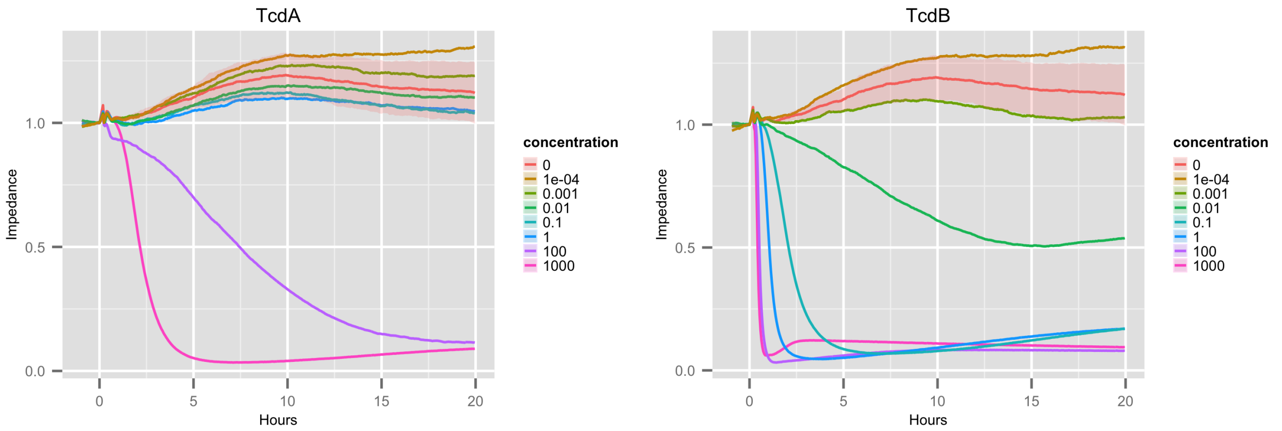

Figure S15: CHO cells treated with TcdA or TcdB

4.3 IMCE cells

```
subset = normalize_toxin(select(wells, "IMCE"), xlim = c(-1, 20))
p1 = plot(select(subset, "TcdA"), title = "TcdA")
p2 = plot(select(subset, "TcdB"), title = "TcdB")
grid.arrange(p1, p2, nrow = 1)
```

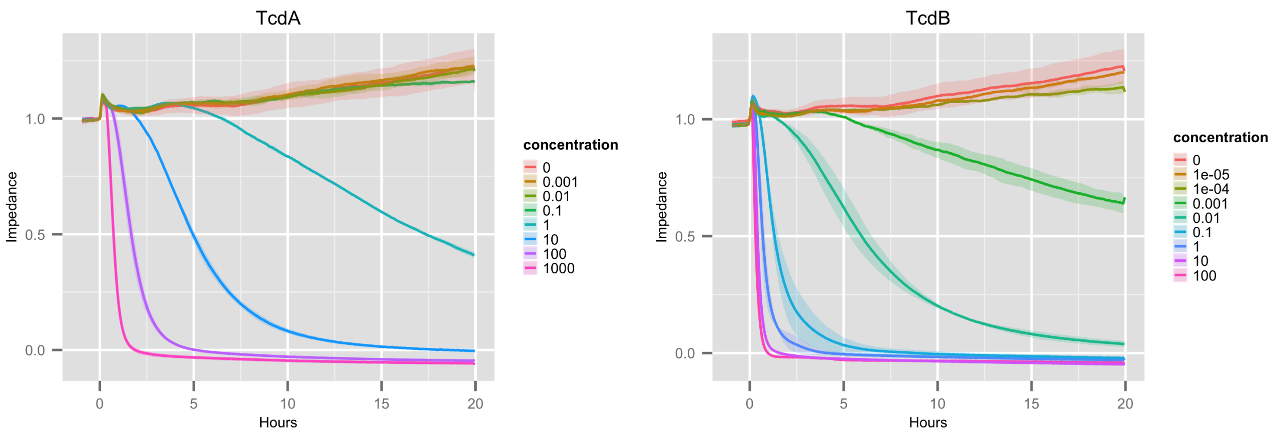

Figure S16: IMCE cells treated with TcdA or TcdB

4.4 HUVECs

```
subset = normalize_toxin(select(wells, "HUVEC"))
p1 = plot(select(subset, "TcdA"), xlim = c(-1, 20), title = "TcdA")
p2 = plot(select(subset, "TcdB"), xlim = c(-1, 5), title = "TcdB")
grid.arrange(p1, p2, nrow = 1)
```

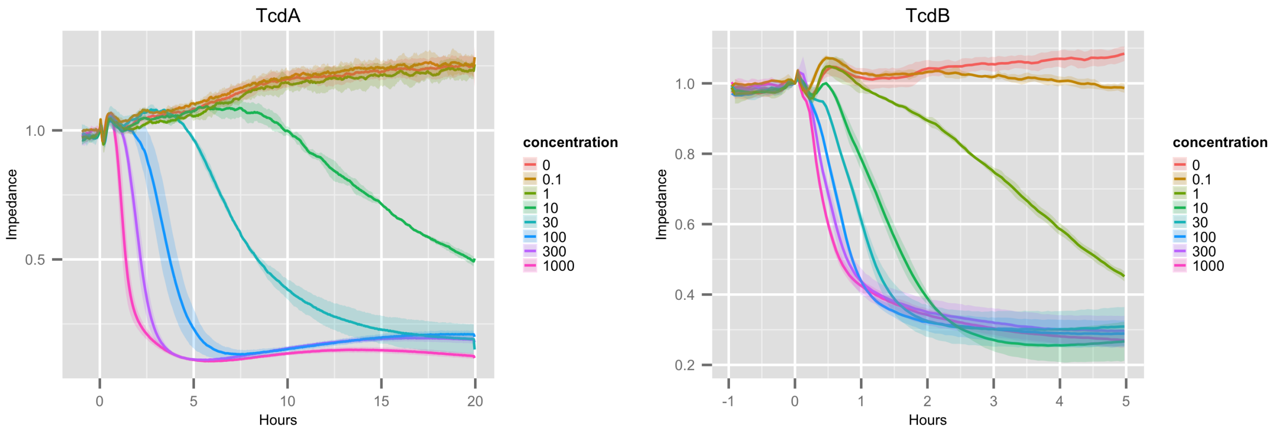

Figure S17: HUVECs treated with TcdA or TcdB

4.5 T84 cells

```
subset = normalize_toxin(select(wells, "T84"), xlim = c(-1, 20))
p1 = plot(select(subset, "TcdA"), title = "TcdA")
p2 = plot(select(subset, "TcdB"), title = "TcdB")
grid.arrange(p1, p2, nrow = 1)
```

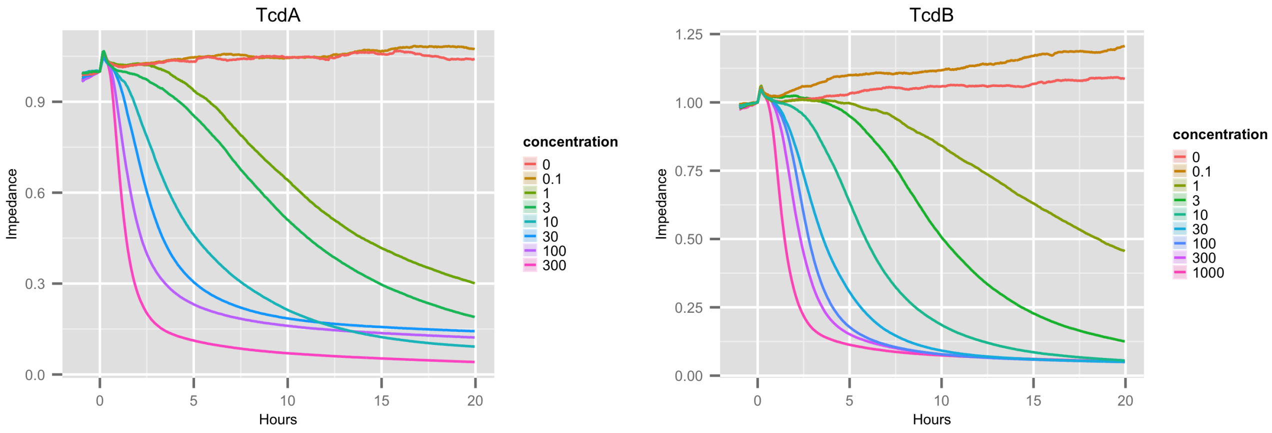

Figure S18: T84 cells treated with TcdA or TcdB

4.6 J774 cells

TcdA and TcdB were added to confluent J774 cells.

```
subset = normalize_toxin(select(wells, file = c("J774-a.txt", "J774-b.txt")))
p1 = plot(select(subset, "TcdA"), xlim = c(-1, 40), title = "TcdA")
p2 = plot(select(subset, "TcdB"), xlim = c(-1, 40), title = "TcdB")
grid.arrange(p1, p2, nrow = 1)
```

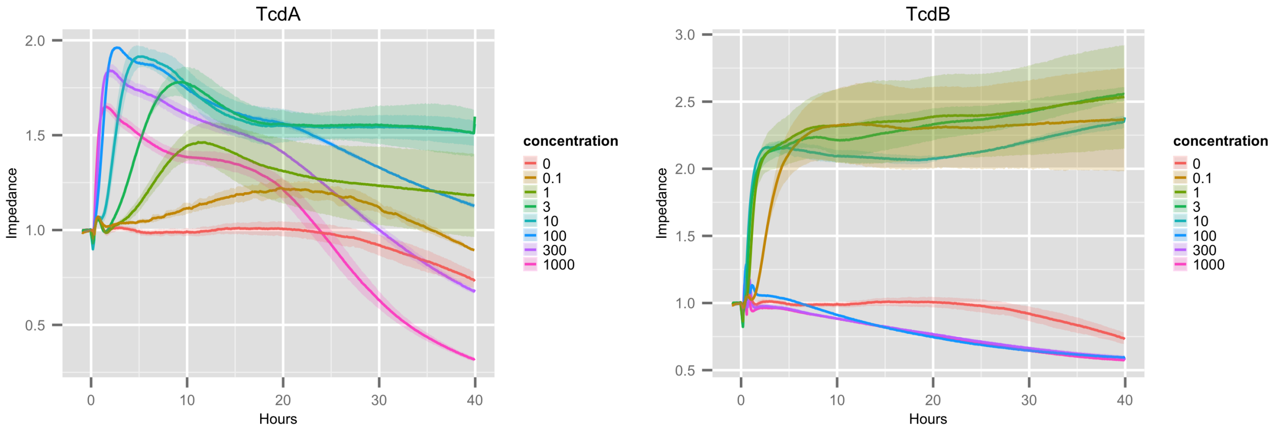

Figure S19: J774 cells treated with TcdA or TcdB

Since the lowest TcdB concentration tested in the first experiment still had an effect, a fuller concentration-response experiment was performed with TcdB. The lowest concentration to cause a response was approximately 1 pg/ml.

```
subset = normalize_toxin(select(wells, file = "J774-2.txt"))
plot(subset, xlim = c(-1, 24), title = "TcdB")
```

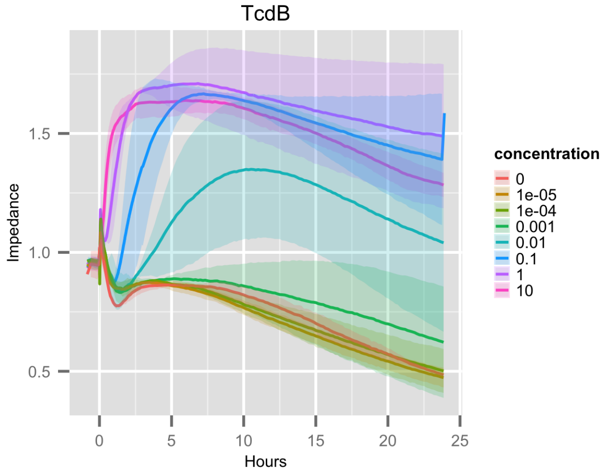

Figure S20: J774 cells treated with TcdB

Like with HCT8 cells, gdTcdB was combined with TcdA and TcdB to see if it delayed their effects. No delay was clearly observed, yet gdTcdB alone elicited a distinct response at 100 ng/ml (but not 0.1 or 1 ng/ml).

```
subset = select(wells, file = c("J774-3a.txt", "J774-3b.txt"))
subset = normalize_toxin(subset, xlim = c(-1, 10))
p1 = plot(select(subset, "gdTcdB & !(TcdA | TcdB)"), title = "gdTcdB")
p2 = plot(select(subset, "TcdA"), title = "TcdA")
p3 = plot(select(subset, "TcdB"), title = "TcdB")
grid.arrange(p1, p2, p3, nrow = 2)
```

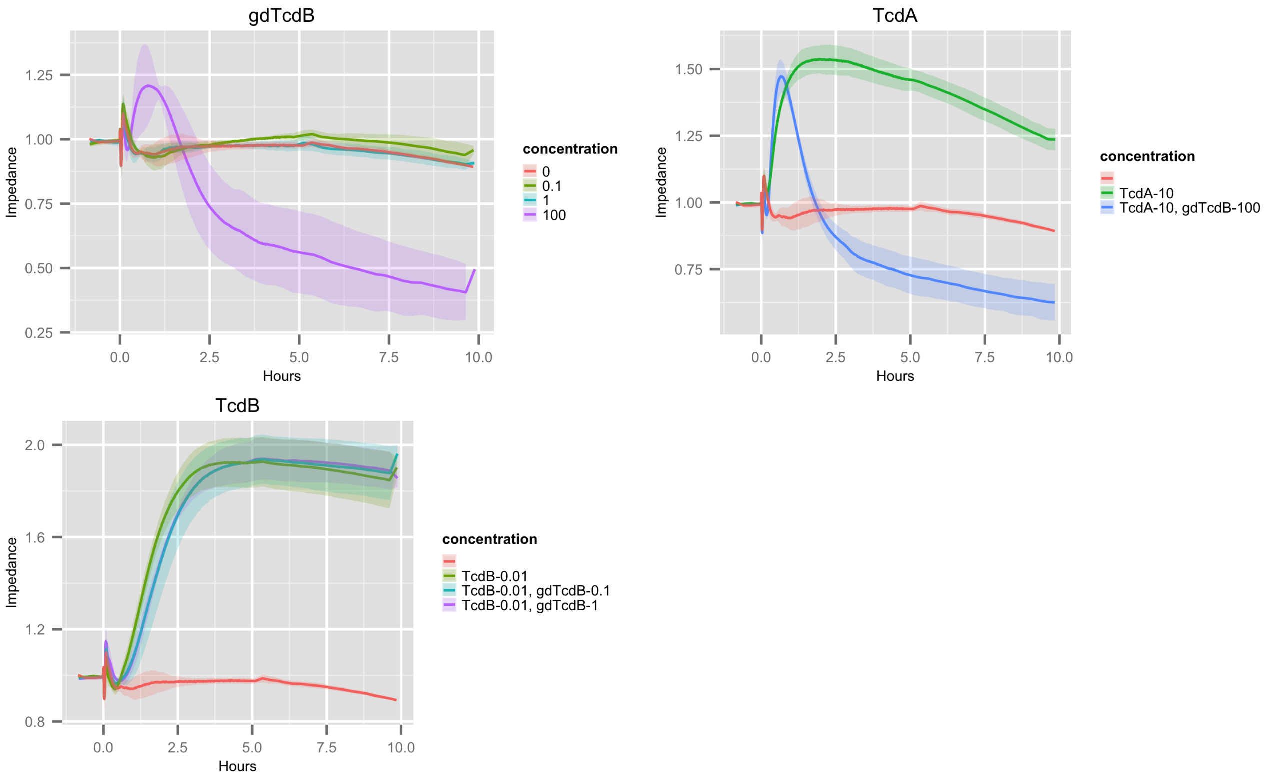

Figure S21: J774 cells treated with TcdA, TcdB, and/or gdTcdB

In the next experiment, toxin was added to subconfluent cells for the sake of easier imaging of individual cells. The impedance increases much more than with confluent cells likely because the spreading of macrophages is more obvious when fewer cells are already covering the electrodes before the toxin is added. Again, TcdB and gdTcdB at 100 ng/ml induced a temporary increase in impedance followed by a long decrease.

```
subset = normalize_toxin(select(wells, file = "J774-4.txt"), xlim = c(-1, 24))
p1 = plot(select(subset, "gdTcdB & !(TcdA | TcdB)"), title = "gdTcdB")
p2 = plot(select(subset, "TcdA"), title = "TcdA")
p3 = plot(select(subset, "TcdB"), title = "TcdB")
grid.arrange(p1, p2, p3, nrow = 2)
```

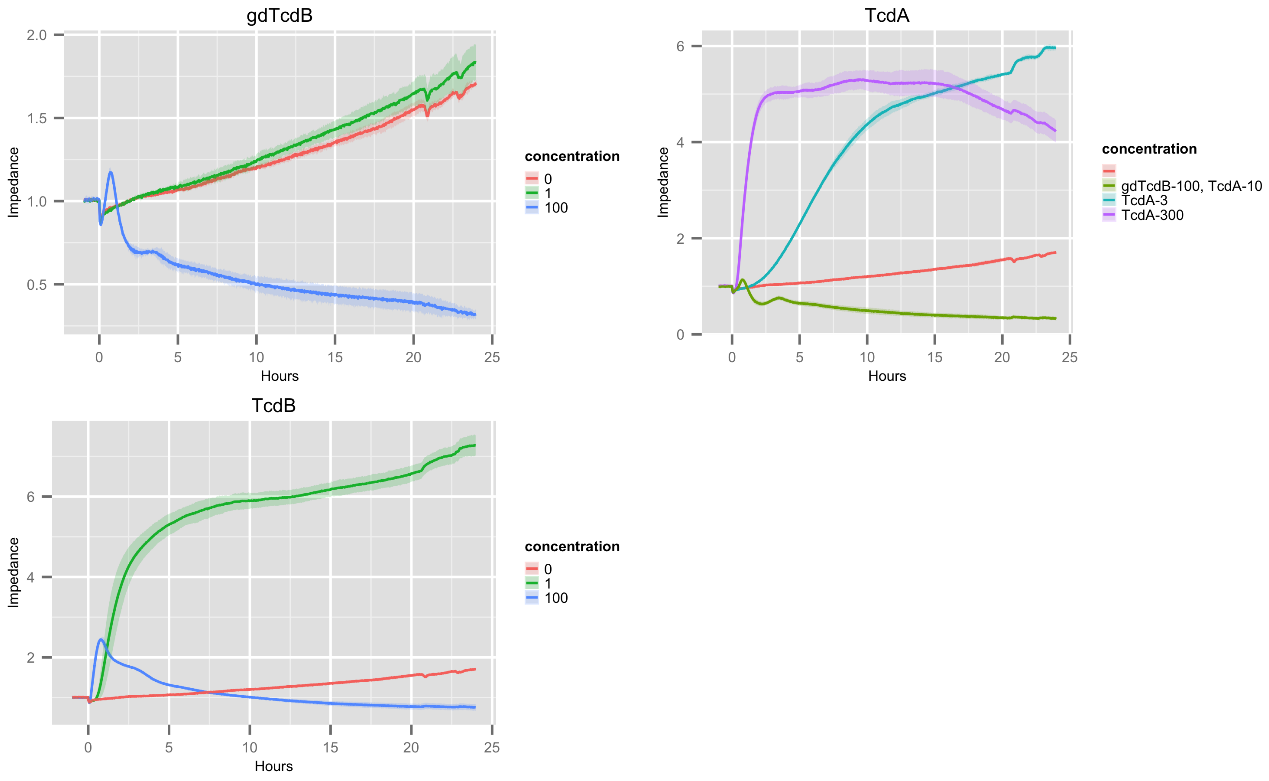

Figure S22: J774 cells treated with TcdA, TcdB, and/or gdTcdB

For the previous experiment, images were taken of replicate wells and are shown below.

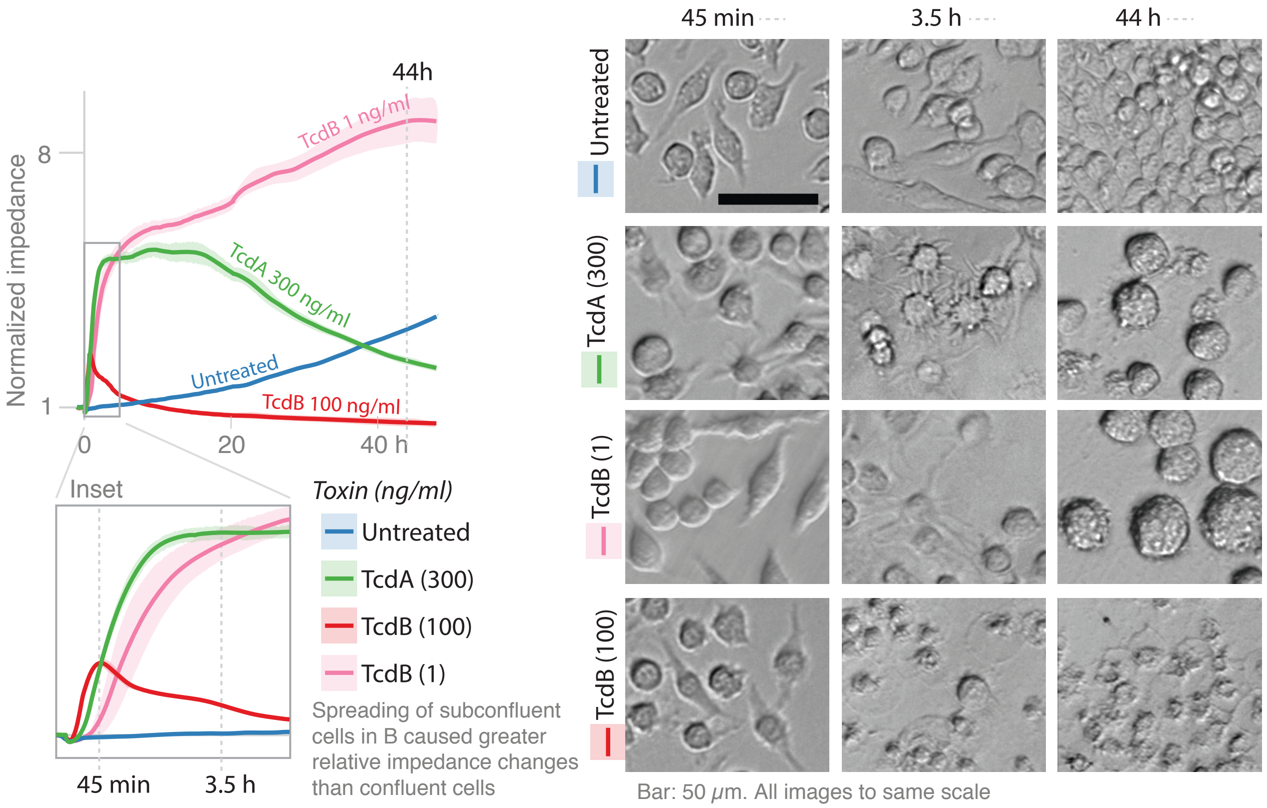

Figure S23: Structural changes of J774 cells after toxin addition

Different combinations of gdTcdB and TcdA or TcdB were added to cells to explore other potential interactions. None were found; the effects appeared to be additive.

```
subset = normalize_toxin(select(wells, file = "J774-5.txt"), xlim = c(-1, 24))
p1 = plot(select(subset, "gdTcdB & !(TcdA | TcdB)"), title = "gdTcdB")
p2 = plot(select(subset, "TcdA"), title = "TcdA")
p3 = plot(select(subset, "TcdB"), title = "TcdB")
grid.arrange(p1, p2, p3, nrow = 2)
```

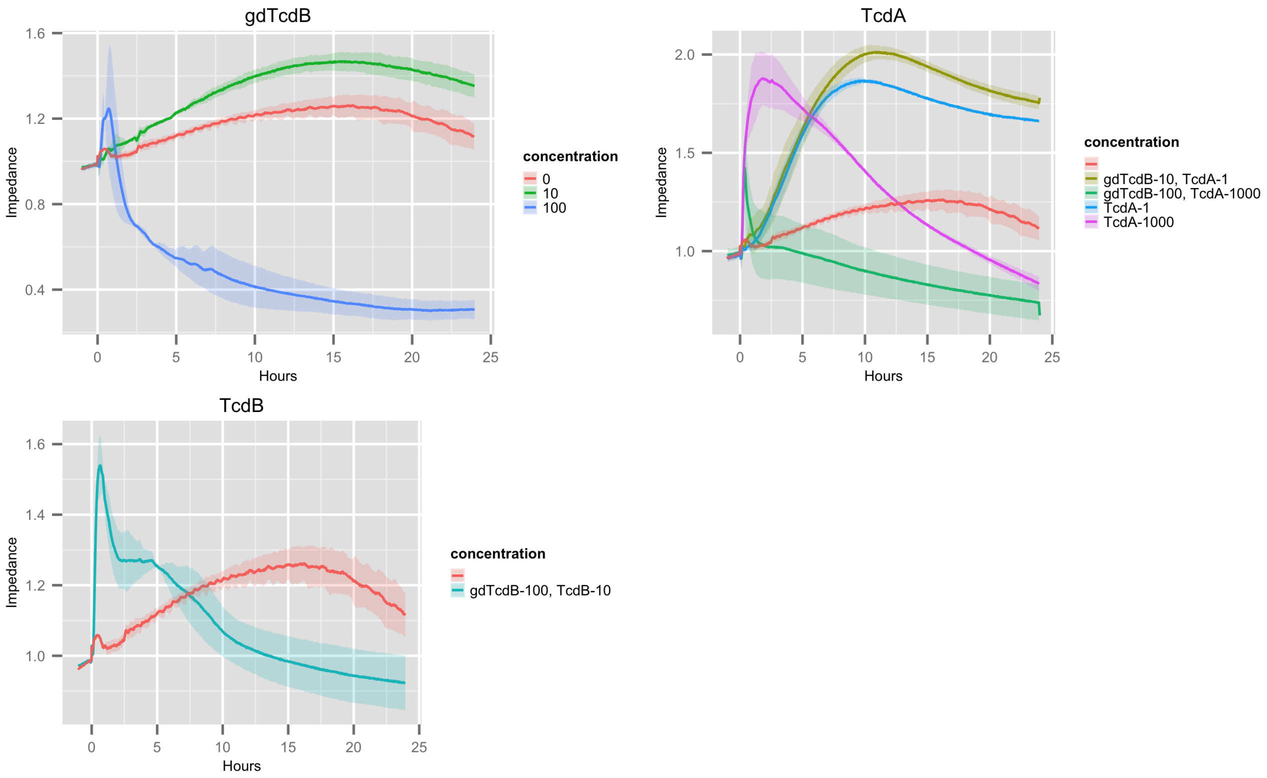

Figure S24: J774 cells treated with TcdA, TcdB, and/or gdTcdB

A sixth experiment was carried out with J774 cells to confirm the previous findings. TcdB and gdTcdB at 100 ng/ml decreased J774 impedance; TcdA and TcdB at lower concentrations increased impedance; and gdTcdB did not clearly alter the affects of TcdA or TcdB.

```
subset = normalize_toxin(select(wells, file = "J774-6.txt"), xlim = c(-1, 24))
p1 = plot(select(subset, "gdTcdB & !(TcdA | TcdB)"), title = "gdTcdB")
p2 = plot(select(subset, "TcdA[1] | gdTcdB[10] & !TcdB | (TcdA[100] & !gdTcdB)"),
    title = "TcdA 1 ng/ml")
p3 = plot(select(subset, "TcdA & !TcdA[10] & !gdTcdB"), title = "TcdA")
p4 = plot(select(subset, "TcdB[0.1] | gdTcdB[1-10] & !TcdA"), xlim = c(0, 5),
    title = "TcdB 0.1 ng/ml")
p5 = plot(select(subset, "TcdB & !gdTcdB"), title = "TcdB")
grid.arrange(p1, p2, p3, p4, p5, nrow = 3)
```

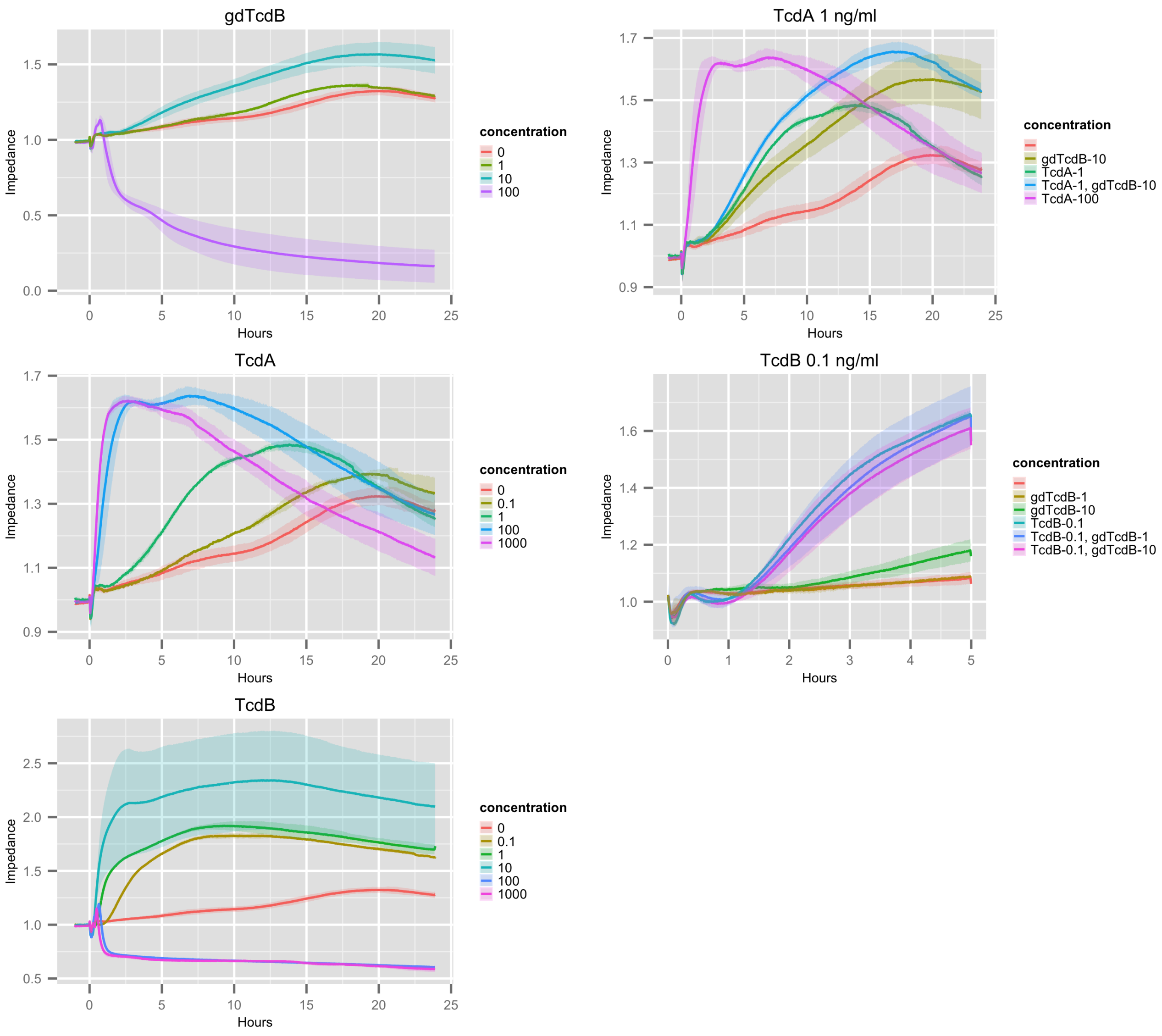

Figure S25: J774 cells treated with TcdA, TcdB, and/or gdTcdB

4.7 PMN leukocytes

Consistent response profiles of PMNs to TcdA or TcdB could not be obtained. This is likely because unstimulated PMNs are not adherent and so changes in shape are difficult to measure by impedance.

In the first experiment, only TcdA at 1,000 ng/ml was clearly different than control cells. Instead of normalizing the impedance at the time toxin was added, the change in impedance from the time of toxin addition is shown.

```
subset = select(wells, file = c("PMN-2a.txt", "PMN-2b.txt"))
subset2 = transform(subset, c("tcenter", "level", "slice"), ID = "toxinAdd",
    xlim = c(-1, 24))
p1 = plot(select(subset2, "TcdA"), title = "TcdA")
p2 = plot(select(subset2, "TcdB"), title = "TcdB")
grid.arrange(p1, p2, nrow = 1)
```

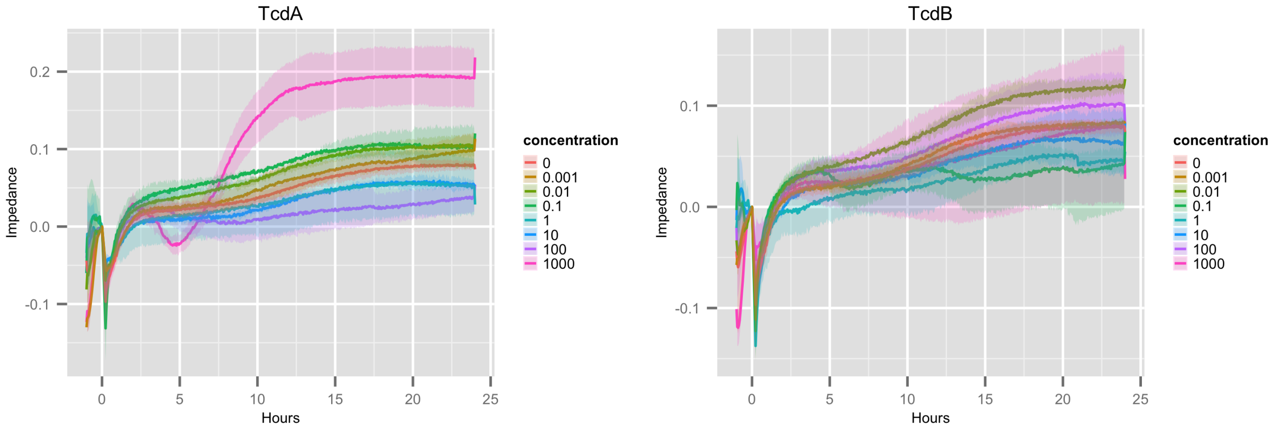

Figure S26: PMN leukocytes treated with TcdA or TcdB

In the next experiment, cells were seeded in the presence of 100 ng/ml (9.01 nM) of human recombinant IL-8 in an attempt to increase impedance before adding toxin.

```
subset = select(wells, file = c("PMN-a.txt", "PMN-b.txt"))
subset2 = transform(subset, c("tcenter", "level"), ID = "toxinAdd")
p1 = plot(select(subset2, "!TcdB"), xlim = c(-1, 24), title = "TcdA")
p2 = plot(select(subset2, "!TcdA"), xlim = c(-1, 10), title = "TcdB")
grid.arrange(p1, p2, nrow = 1)
```

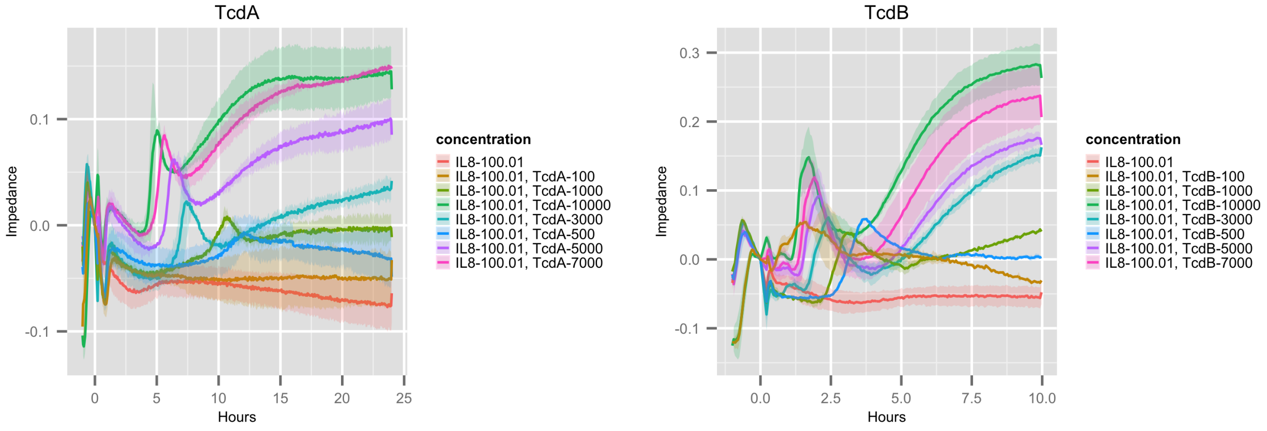

Figure S27: PMN leukocytes treated with IL-8 and then TcdA or TcdB

In another experiment, cells were seeded in the presence of 25 nM (277.45 ng/ml) of IL-8. The results were not consistent with the previous experiment with IL-8.

```
subset = select(wells, file = "PMN-3.txt")
subset2 = transform(subset, c("tcenter", "level", "slice"), ID = "toxinAdd",
    xlim = c(-1, 24))
p1 = plot(select(subset2, "(TcdA & IL8) | (IL8 & !TcdA & !TcdB)"), title = "TcdA+IL8")
p2 = plot(select(subset2, "TcdA & !IL8"), title = "TcdA")
p3 = plot(select(subset2, "(TcdB & IL8) | (IL8 & !TcdA & !TcdB)"), replicates = FALSE,
    title = "TcdB+IL8")
p4 = plot(select(subset2, "TcdB & !IL8"), title = "TcdB")
grid.arrange(p1, p2, p3, p4, nrow = 2)
```

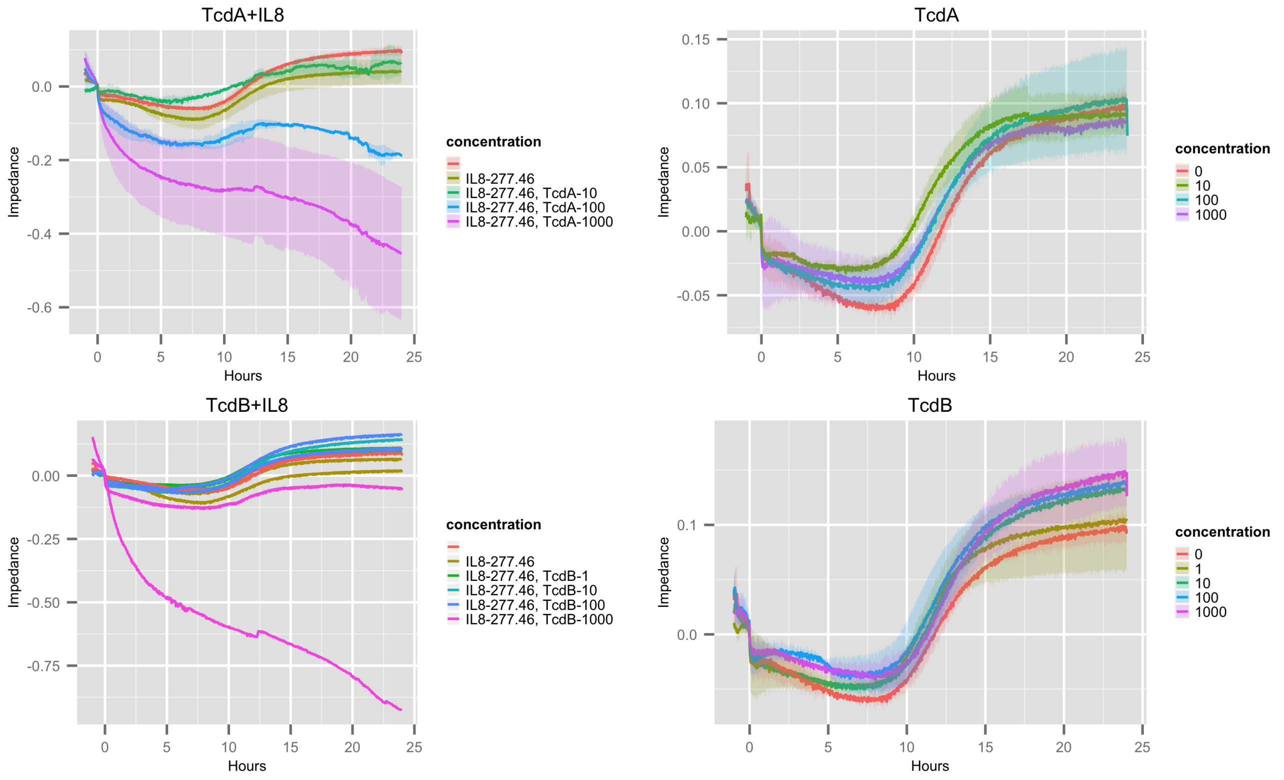

Figure S28: PMN leukocytes treated with IL-8 and then TcdA or TcdB

Again, cells were seeded in the presence of 25 nM (277.45 ng/ml) of IL-8. The machine temporarily stopped making impedance measurements from approximately five to 20 hours.

```
subset = select(wells, file = "PMN-4.txt")
subset2 = transform(subset, c("tcenter", "level", "slice"), ID = "toxinAdd",
    xlim = c(-1, 36))
p1 = plot(select(subset2, "(TcdA & IL8) | (IL8 & !TcdA & !TcdB)"), title = "TcdA+IL8")
p2 = plot(select(subset2, "TcdA & !IL8"), title = "TcdA")
p3 = plot(select(subset2, "(TcdB & IL8) | (IL8 & !TcdA & !TcdB)"), replicates = FALSE,
    title = "TcdB+IL8")
p4 = plot(select(subset2, "TcdB & !IL8"), title = "TcdB")
grid.arrange(p1, p2, p3, p4, nrow = 2)
```

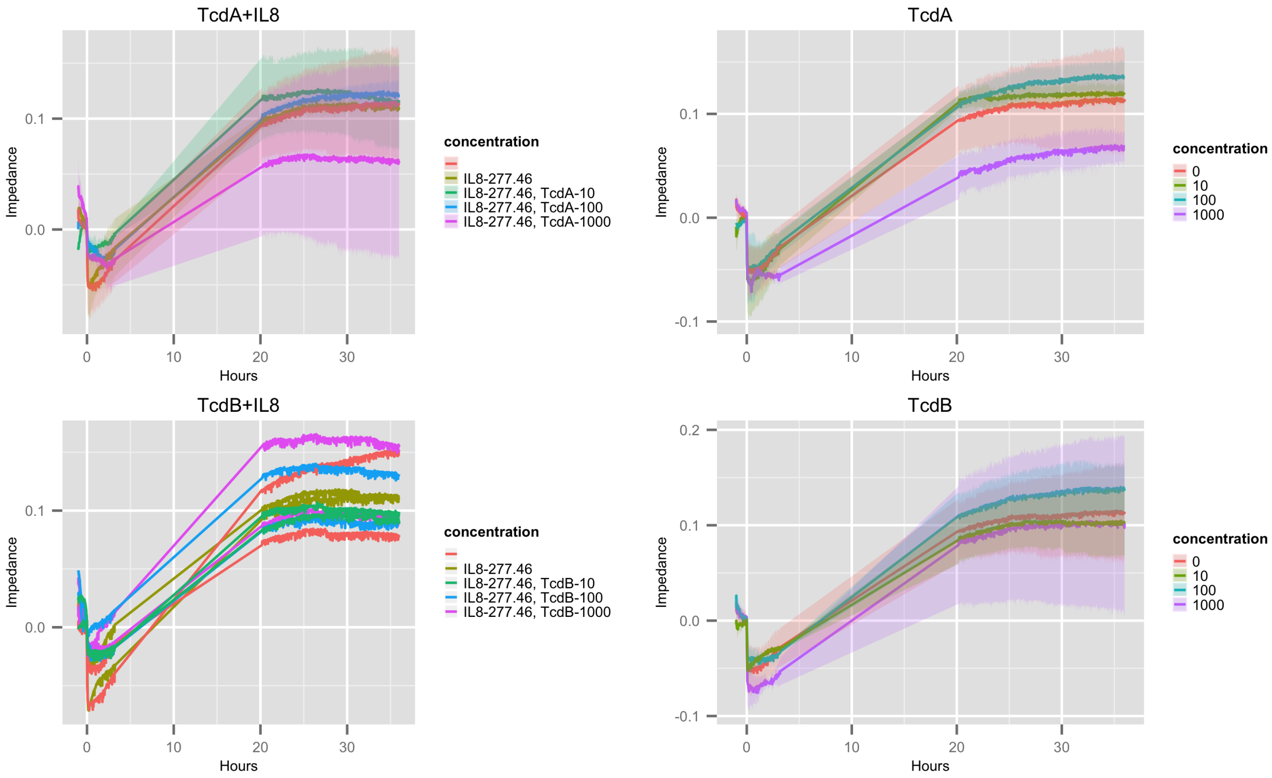

Figure S29: PMN leukocytes treated with IL-8 and then TcdA or TcdB

4.8 Plate Layouts

Below are the layouts of all of the plates used.

```r
fwells = split(wells, filename(wells))
fwells = fwells[sort(names(fwells))]
latex.code = lapply(fwells, latex_layout, ID = "toxinAdd", floating = FALSE,
    scalebox = 0.6)
do.call(cat, c("\\begin{adjustwidth}{0in}{3in}{", latex.code, "}\\end{adjustwidth}"))
```

	1	2
A	TcdA-1000	TcdB-1000
B		TcdB-100
C	TcdA-100	TcdB-1
D	TcdA-1	TcdB-0.1
E	TcdA-0.1	TcdB-0.01
F	TcdA-0.01	
G	TcdA-0.001	TcdB-0.001
H	TcdA-1e-04	TcdB-1e-04

CHO.txt

	1	2
A	TcdB-1, gdTcdB-100	TcdB-1, gdTcdB-100
B	TcdB-1, gdTcdB-10	TcdB-1, gdTcdB-10
C	TcdA-100, gdTcdB-1000	TcdA-100, gdTcdB-1000
D	TcdB-1	TcdB-1
E	TcdA-100	TcdA-100
F	gdTcdB-1000	gdTcdB-1000
G	gdTcdB-100	gdTcdB-100
H		

HCT8-2a.txt

	3	4
A	TcdB-1, gdTcdB-100	TcdB-1, gdTcdB-100
B	TcdB-1, gdTcdB-10	TcdB-1, gdTcdB-10
C	TcdA-100, gdTcdB-1000	TcdA-100, gdTcdB-1000
D	TcdB-1	TcdB-1
E	TcdA-100	TcdA-100
F	gdTcdB-1000	gdTcdB-1000
G	gdTcdB-100	gdTcdB-100
H		

HCT8-2b.txt

	3	4	5	6
A	TcdA-100, gdTcdB-1000		TcdA-100	gdTcdB-10
B	gdTcdB-100	TcdA-100	TcdA-100, gdTcdB-1000	TcdAup-100
C	TcdA-100	gdTcdB-100		gdTcdB-10
D		TcdA-100, gdTcdB-1000	gdTcdB-100	TcdAup-100
E	TcdB-1	gdTcdB-1000	TcdB-1, gdTcdB-10	gdTcdB-10
F	TcdB-1, gdTcdB-100	TcdB-1, gdTcdB-10	TcdB-1	TcdAother-100
G	TcdB-1, gdTcdB-10	TcdB-1, gdTcdB-100	gdTcdB-1000	TcdAother-100
H	gdTcdB-1000	TcdB-1	TcdB-1, gdTcdB-100	

HCT8-3.txt

	1	2	3	4
A	TcdA-10	TcdB-10	TcdB-100, gdTcdB-1000	TcdB-100, gdTcdB-1000
B	TcdA-1000	TcdA-1000	TcdB-100	TcdB-10
C			TcdA-10, gdTcdB-100	TcdB-100, gdTcdB-100
D	gdTcdB-1000	gdTcdB-1000	TcdA-100, gdTcdB-100	TcdA-1000, gdTcdB-1000
E	gdTcdB-100	TcdA-100	TcdA-1000, gdTcdB-1000	
F	TcdB-10, gdTcdB-100	gdTcdB-100	TcdA-10, gdTcdB-100	TcdA-100, gdTcdB-1000
G	TcdB-10, gdTcdB-100	TcdA-100	TcdB-10, gdTcdB-1000	TcdA-100, gdTcdB-1000
H	TcdB-10, gdTcdB-1000	TcdB-100	TcdA-100, gdTcdB-100	TcdB-100, gdTcdB-100

HCT8-4.txt

	3	4
A	TcdA-100	TcdA-100
B	NA	NA
C	TcdB-100	
D	TcdA-500	TcdB-500
E	TcdA-100	TcdB-100
F	TcdA-10	TcdB-10
G	TcdA-1	TcdB-1
H	TcdA-0.1	TcdB-0.1

HCT8.txt

	3	4
A	TcdA-1000	TcdB-1000
B	TcdA-300	TcdB-300
C	TcdA-100	TcdB-100
D		
E	TcdA-30	TcdB-30
F	TcdA-10	TcdB-10
G	TcdA-1	TcdB-1
H	TcdA-0.1	TcdB-0.1

HUVEC-a.txt

	5	6
A	TcdA-1000	TcdB-1000
B	TcdA-300	TcdB-300
C	TcdA-100	TcdB-100
D		
E	TcdA-30	TcdB-30
F	TcdA-10	TcdB-10
G	TcdA-1	TcdB-1
H	TcdA-0.1	TcdB-0.1

HUVEC-b.txt

	3	4	5	6
A	TcdA-1000	TcdB-100	TcdA-1000	TcdB-10
B	TcdA-100	TcdB-10	TcdA-100	TcdB-1
C		TcdB-1		TcdB-0.1
D	TcdA-10	TcdB-0.1	TcdA-10	TcdB-0.01
E	TcdA-1	TcdB-0.01	TcdA-1	TcdB-0.001
F	TcdA-0.1	TcdB-0.001	TcdA-0.1	TcdB-1e-04
G	TcdA-0.01	TcdB-1e-04	TcdA-0.01	TcdB-1e-05
H	TcdA-0.001	TcdB-1e-05	TcdA-0.001	

IMCE.txt

	5	6
A	10	10
B	1	1
C	0.1	0.1
D	0.01	0.01
E	0.001	0.001
F	1e-04	1e-04
G	1e-05	1e-05
H	0	0

J774-2.txt

	1	2
A	TcdB-0.01, gdTcdB-1	TcdB-0.01, gdTcdB-1
B	TcdB-0.01, gdTcdB-0.1	TcdB-0.01, gdTcdB-0.1
C	TcdA-10, gdTcdB-100	TcdA-10, gdTcdB-100
D	TcdB-0.01	TcdB-0.01
E	TcdA-10	TcdA-10
F	gdTcdB-100	gdTcdB-100
G	gdTcdB-1	gdTcdB-1
H	gdTcdB-0.1	

J774-3a.txt

	3	4
A	TcdB-0.01, gdTcdB-1	TcdB-0.01, gdTcdB-1
B	TcdB-0.01, gdTcdB-0.1	TcdB-0.01, gdTcdB-0.1
C	TcdA-10, gdTcdB-100	TcdA-10, gdTcdB-100
D	TcdB-0.01	TcdB-0.01
E	TcdA-10	TcdA-10
F	gdTcdB-100	
G		gdTcdB-1
H	gdTcdB-0.1	gdTcdB-0.1

J774-3b.txt

	1	2
A	TcdB-1	TcdB-1
B	TcdB-100	TcdB-100
C		
D	gdTcdB-1	gdTcdB-1
E	gdTcdB-100	gdTcdB-100
F	TcdA-3	TcdA-3
G	TcdA-300	TcdA-300
H	gdTcdB-100, TcdA-10	gdTcdB-100, TcdA-10

J774-4.txt

	1	2
A	gdTcdB-100, TcdB-10	gdTcdB-100, TcdB-10
B	gdTcdB-10, TcdA-1	gdTcdB-10, TcdA-1
C	gdTcdB-100, TcdA-1000	gdTcdB-100, TcdA-1000
D	TcdA-1	TcdA-1
E	TcdA-1000	TcdA-1000
F	gdTcdB-10	gdTcdB-10
G	gdTcdB-100	gdTcdB-100
H		

J774-5.txt

	1	2	3	4	5	6
A						
B	gdTcdB-10	gdTcdB-10	TcdB-0.1, gdTcdB-1	TcdB-0.1, gdTcdB-1	gdTcdB-100	gdTcdB-100
C	TcdA-1, gdTcdB-10	TcdA-1, gdTcdB-10	TcdB-0.1, gdTcdB-10	TcdB-0.1, gdTcdB-10	TcdB-0.1, gdTcdB-1	TcdB-0.1, gdTcdB-1
D	TcdA-0.1	TcdA-0.1	TcdB-0.1	TcdB-0.1	TcdB-0.1, gdTcdB-10	TcdB-0.1, gdTcdB-10
E	TcdA-1	TcdA-1	TcdB-1	TcdB-1	TcdA-1, gdTcdB-10	TcdA-1, gdTcdB-10
F	TcdA-10	TcdA-10	TcdB-10	TcdB-10	gdTcdB-1	gdTcdB-1
G	TcdA-100	TcdA-100	TcdB-100	TcdB-100	gdTcdB-10	gdTcdB-10
H	TcdA-1000	TcdA-1000	TcdB-1000	TcdB-1000	gdTcdB-100	gdTcdB-100

J774-6.txt

	1	2
A	TcdA-1000	TcdB-1000
B	TcdA-300	TcdB-300
C		
D	TcdA-100	TcdB-100
E	TcdA-10	TcdB-10
F	TcdA-3	TcdB-3
G	TcdA-1	TcdB-1
H	TcdA-0.1	TcdB-0.1

J774-a.txt

	5	6
A	TcdA-1000	TcdB-1000
B	TcdA-300	TcdB-300
C		Other-100
D	TcdA-100	TcdB-100
E	TcdA-10	TcdB-10
F	TcdA-3	TcdB-3
G	TcdA-1	TcdB-1
H	TcdA-0.1	TcdB-0.1

J774-b.txt

	3	4
A	TcdA-1000	TcdB-1000
B	TcdA-100	TcdB-100
C	TcdA-10	TcdB-10
D	TcdA-1	TcdB-1
E	TcdA-0.1	TcdB-0.1
F		TcdB-0.01
G	TcdA-0.01	TcdB-0.001
H	TcdA-0.001	NA

PMN-2a.txt

	5	6
A	TcdA-1000	TcdB-1000
B	TcdA-100	TcdB-100
C	TcdA-10	TcdB-10
D	TcdA-1	TcdB-1
E	TcdA-0.1	TcdB-0.1
F		TcdB-0.01
G	TcdA-0.01	TcdB-0.001
H	TcdA-0.001	

PMN-2b.txt

	3	4	5	6
A	IL8-277.46, TcdB-1	IL8-277.46, TcdB-1	TcdB-1	TcdB-1
B	IL8-277.46, TcdB-10	IL8-277.46, TcdB-10	TcdB-10	TcdB-10
C	IL8-277.46	IL8-277.46		
D	IL8-277.46, TcdB-100	IL8-277.46, TcdB-100	TcdB-100	TcdB-100
E	IL8-277.46, TcdB-1000	IL8-277.46, TcdB-1000	TcdB-1000	TcdB-1000
F	IL8-277.46, TcdA-1000	IL8-277.46, TcdA-1000	TcdA-1000	TcdA-1000
G	IL8-277.46, TcdA-100	IL8-277.46, TcdA-100	TcdA-100	TcdA-100
H	IL8-277.46, TcdA-10	IL8-277.46, TcdA-10	TcdA-10	TcdA-10

PMN-3.txt

	1	2	3	4
A	IL8-277.46, TcdA-10	IL8-277.46, TcdA-10	TcdA-10	TcdA-10
B	IL8-277.46, TcdA-100	IL8-277.46, TcdA-100	TcdA-100	TcdA-100
C	IL8-277.46	IL8-277.46		
D	IL8-277.46, TcdA-1000	IL8-277.46, TcdA-1000	TcdA-1000	TcdA-1000
E	IL8-277.46, TcdB-1000	IL8-277.46, TcdB-1000	TcdB-1000	TcdB-1000
F	IL8-277.46, TcdB-100	IL8-277.46, TcdB-100	TcdB-100	TcdB-100
G	IL8-277.46, TcdB-10	IL8-277.46, TcdB-10	TcdB-10	TcdB-10

PMN-4.txt

	1	2
A	IL8-100.01, TcdA-10000	IL8-100.01, TcdB-10000
B	IL8-100.01, TcdA-7000	IL8-100.01, TcdB-7000
C	IL8-100.01, TcdA-5000	IL8-100.01, TcdB-5000
D	IL8-100.01	IL8-100.01
E	IL8-100.01, TcdB-3000	IL8-100.01, TcdA-3000
F	IL8-100.01, TcdB-1000	IL8-100.01, TcdA-1000
G	IL8-100.01, TcdB-500	IL8-100.01, TcdA-500
H	IL8-100.01, TcdB-100	IL8-100.01, TcdA-100

PMN-a.txt

	3	4
A	IL8-100.01, TcdA-10000	IL8-100.01, TcdB-10000
B	IL8-100.01, TcdA-7000	IL8-100.01, TcdB-7000
C	IL8-100.01, TcdA-5000	IL8-100.01, TcdB-5000
D	IL8-100.01	IL8-100.01
E	IL8-100.01, TcdB-3000	IL8-100.01, TcdA-3000
F	IL8-100.01, TcdB-1000	IL8-100.01, TcdA-1000
G	IL8-100.01, TcdB-500	IL8-100.01, TcdA-500
H	IL8-100.01, TcdB-100	IL8-100.01, TcdA-100

PMN-b.txt

	3	4
A	rTcdB-1000	TcdB-1000
B	rTcdB-100	TcdB-100
C	rTcdB-10	TcdB-10
D	rTcdB-1	TcdB-1
E		
F	rTcdB-0.1	TcdB-0.1
G	rTcdB-0.01	TcdB-0.01
H	rTcdB-0.001	TcdB-0.001

rHCT8.txt

	3	4
A	NA	NA
B	300	1
C	100	0.1
D	30	NA
E	10	0
F	3	NA
G	NA	NA
H	NA	NA

T84-a.txt

	5	6
A	NA	NA
B	NA	1000
C	3	300
D	0	100
E	1	30
F	0.1	10
G	NA	NA
H	NA	NA

T84-b.txt
